# The membrane electric field regulates the PIP_2_-binding site to gate the KCNQ1 channel

**DOI:** 10.1073/pnas.2301985120

**Published:** 2023-05-16

**Authors:** Venkata Shiva Mandala, Roderick MacKinnon

**Affiliations:** ^a^Laboratory of Molecular Neurobiology and Biophysics, The Rockefeller University, New York, NY 10065; ^b^HHMI, The Rockefeller University, New York, NY 10065

**Keywords:** KCNQ1 channel, K_v_7.1 channel, voltage sensor, cryo-EM, membrane potential

## Abstract

Voltage-dependent ion channels underlie the propagation of action potentials and other forms of electrical activity in cells. In these proteins, voltage sensor domains (VSDs) regulate opening and closing of the pore through the displacement of their positive-charged S4 helix in response to the membrane voltage. The movement of S4 at hyperpolarizing membrane voltages in some channels is thought to directly clamp the pore shut through the S4–S5 linker helix. The KCNQ1 channel (also known as K_v_7.1), which is important for heart rhythm, is regulated not only by membrane voltage but also by the signaling lipid phosphatidylinositol 4,5-bisphosphate (PIP_2_). KCNQ1 requires PIP_2_ to open and to couple the movement of S4 in the VSD to the pore. To understand the mechanism of this voltage regulation, we use cryogenic electron microscopy to visualize the movement of S4 in the human KCNQ1 channel in lipid membrane vesicles with a voltage difference across the membrane, i.e., an applied electric field in the membrane. Hyperpolarizing voltages displace S4 in such a manner as to sterically occlude the PIP_2_-binding site. Thus, in KCNQ1, the voltage sensor acts primarily as a regulator of PIP_2_ binding. The voltage sensors’ influence on the channel’s gate is indirect through the reaction sequence: voltage sensor movement → alter PIP_2_ ligand affinity → alter pore opening.

## Significance

Voltage-gated ion channels underlie electrical signaling in cells. The structures and functions of voltage-dependent K^+^, Na^+^, and Ca^2+^ and transient receptor potential ion channels have been studied extensively since their discovery. Despite these efforts, it is still not well understood how the voltage sensors in these different ion channels change their conformation in response to membrane voltage changes, and how these movements regulate the opening or closing of the channel’s gate. This study presents structures of the human KCNQ1 (K_v_7.1) voltage–dependent and phosphatidylinositol 4,5-bisphosphate (PIP_2_)-dependent K^+^ channel in electrically polarized lipid vesicles using cryogenic electron microscopy, showing how the voltage sensors influence gating indirectly by regulating the ability of PIP_2_ to bind to the channel.

Voltage sensor domains (VSDs) are integral membrane proteins that undergo conformational changes in response to voltage differences across the cell membrane. These domains regulate pore opening and closing in voltage-dependent ion channels (1) and enzymatic activity in voltage-dependent phosphatases (2). Voltage sensors have a conserved structure consisting of four transmembrane (TM) helices (S1 to S4) that form a helical bundle (3–6). The fourth helix, S4, contains a repeated sequence of positive-charged amino acids (typically arginines), every third residue that confers sensitivity to voltage. Inside the lipid bilayer, a gating charge transfer center, composed of aspartate, glutamate, and phenylalanine residues, stabilizes the arginines one at a time as they traverse the hydrophobic core of the membrane (7, 8). The movement of S4 in response to the TM voltage difference is ultimately responsible for the regulation of protein activity. This mechanism underlies the action potential in neurons (1, 9) and the initiation of muscle contraction (4, 10), among other cellular processes.

**Fig. 1.**
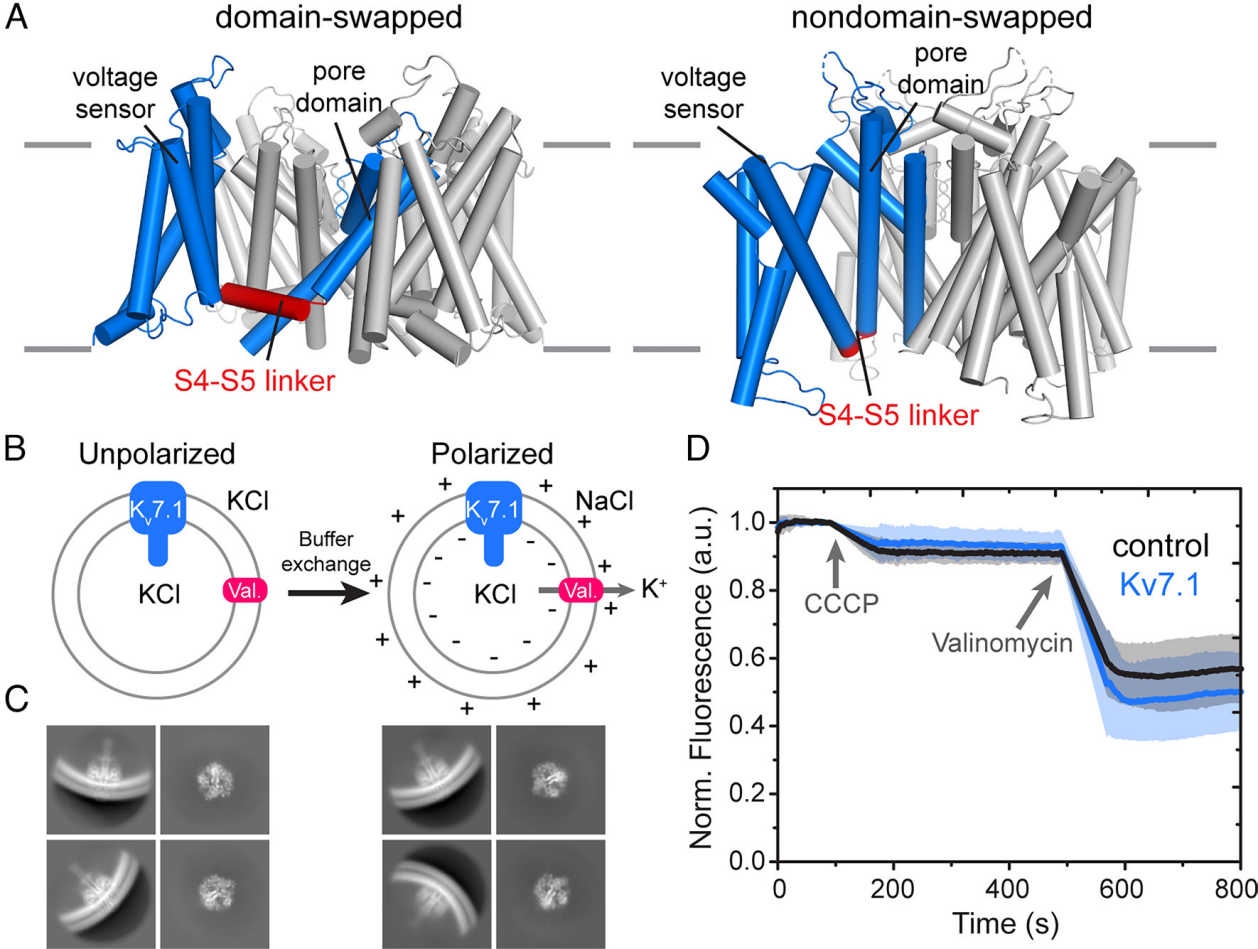
Structures of voltage-dependent ion channels and the preparation of unpolarized and polarized KCNQ1 (K_v_7.1) proteoliposomes. (*A*) The two domain arrangements in voltage-dependent ion channels. Channels are shown with α-helix cylinders and one of the four subunits colored blue and the S4–S5 linker colored red. In domain-swapped channels (*Left*), the VSD of one subunit interacts with the pore domain of an adjacent subunit and is connected to the pore domain through a long interfacial helix––the S4–S5 linker (red). The structure of K_v_1.2 paddle chimera (PDB ID: 2R9R) (7) is shown as an example. In nondomain-swapped channels (*Right*), the VSD interacts with the pore domain of the same subunit through a short S4–S5 loop. The Eag channel (PDB ID: 8EOW) (20) is shown here as an example. (*B*) Schematic of the protocol used to obtain polarized vesicles for cryo-EM analysis. K_v_7.1 is reconstituted into liposomes with symmetrical KCl, and valinomycin (val.) is added to mediate K^+^-flux. The external KCl is exchanged for NaCl using a buffer-exchange column. Potassium efflux through valinomycin generates a potential difference across the membrane such that the inside of the vesicle is negative with respect to the outside. Unpolarized and polarized vesicles containing K_v_7.1 were frozen on a holey carbon grid for structure determination. (*C*) Two-dimensional class-averages of membrane-embedded Kv7.1 in unpolarized (*Left*) and polarized (*Right*) vesicles from cryo-EM. (*D*) Liposome-based flux assay to test polarization of vesicles. Recordings (n = 5, mean ± SD) were made using empty vesicles (black) or vesicles with K_v_7.1 (blue). Addition of the H^+^-ionophore CCCP allows entry of protons, which is detected by quenching of the fluorescent reporter ACMA. Protons enter when the K^+^ ionophore valinomycin is added.

While the structure of a VSD is highly conserved across all voltage-dependent ion channels, there are two configurations for VSD attachment to the pore of the channel (formed by the S5 and S6 helices). In the so-called domain-swapped channels (Fig. 1*A*), which include voltage-dependent K^+^ (K_v_) channels 1 to 9, Na^+^ (Na_v_) channels, Ca^2+^ (Ca_v_) channels, and most transient receptor potential channels, the VSD of one subunit interacts with the pore domain of an adjacent subunit, connected through a long interfacial helix—the S4–S5 linker (7, 11–16). Meanwhile, in nondomain-swapped channels (Fig. 1*A*) such as K_v_10-12, Slo1, and hyperpolarization-activated cyclic nucleotide-gated (HCN) channels, the VSD contacts the pore domain of the same subunit through a short S4–S5 loop (17–19). This naturally raises the question: how do the conserved VSDs mediate voltage-dependent gating in these two sets of channels with different structures?

We have shown recently that in a nondomain-swapped channel Eag (K_v_10.1) (20), the S4 helix on the cytoplasmic side forms an interfacial helix in the hyperpolarized (i.e., negative voltage inside) conformation, which functions as a constrictive cuff around the pore, preventing it from opening. Domain-swapped channels already have an interfacial S4–S5 linker helix that contacts the S6 helix in the depolarized (i.e., no applied or positive inside voltage) conformation (Fig. 1*A*) (7, 16), suggesting a gating mechanism that is distinct from that in nondomain-swapped channels. In domain-swapped channels, it has been proposed that the displacement of S4 in response to a hyperpolarizing potential moves the S4–S5 linker helix into a position that clamps the pore shut by pushing down on the S6 helical bundle (7, 16). Structures of domain-swapped channels in detergent micelles at zero mV with chemical cross-links, toxins, mutations, and metal affinity bridges thought to mimic the hyperpolarized condition are supportive of this mechanism (21–27). Here, we present a cryo-EM analysis of the domain-swapped human KCNQ1 (K_v_7.1) channel in lipid membrane vesicles with a hyperpolarizing voltage generated across the membrane, illustrating—at least in some domain-swapped channels—a different gating mechanism than previously thought.

## Results

### The Rationale for Polarizing KCNQ1.

The KCNQ1 K_v_ channel, also known as K_v_7.1, is the pore-forming subunit of the slow delayed rectifier potassium channel (*I_KS_*) (28, 29) that plays an important role in the repolarization phase of cardiac action potentials (30, 31). Mutations in the *kcnq1* gene are associated with several congenital cardiac diseases, including long and short QT syndromes as well as familial atrial fibrillation (32). Importantly, KCNQ1 and other K_v_7 members are regulated both by membrane voltage and the signaling lipid phosphatidylinositol 4,5-bisphosphate (PIP_2_) (33–36). The voltage sensors close the channel at hyperpolarizing membrane voltages, while PIP_2_ is required for the channel to open. When PIP_2_ is depleted in the membrane, such as when phospholipase C is activated through stimulation of G_q_-coupled receptors (34, 37), the voltage sensors undergo voltage-dependent conformational changes, but the pore does not open at depolarizing voltages (38, 39). PIP_2_ is thus thought to be required for the coupling of voltage sensor movements to pore opening. In other words, KCNQ1 is thought to act as a ligand-regulated voltage-dependent channel, where the binding of PIP_2_ allows the channel to be gated by the membrane potential.

In the absence of PIP_2_ at zero mV, we would expect a closed pore and depolarized voltage sensors, which is exactly the KCNQ1 structure observed in detergent micelles (12, 40). If we now apply a hyperpolarizing voltage across the membrane, the pore should remain closed, but the voltage sensors should adopt the polarized conformation. Because in this circumstance the voltage sensors do not have to perform mechanical work to close the pore, it should be easier to move the voltage sensors when the pore is already closed (due to the absence of PIP_2_). We note that we exclude the KCNE beta subunits (41) in this study because they are known to modify the voltage sensitivity of KCNQ1 and thus could trap the voltage sensor in a specific conformation (for instance, KCNE3 appears to stabilize the depolarized conformation) (40, 42).

### KCNQ1 Reconstitution and Polarization.

The human KCNQ1 channel was purified as a complex with the structurally obligate subunit calmodulin (40, 43) in the presence of Ca^2+^ and reconstituted into liposomes composed of 90: 5: 5 1-palmitoyl-2-oleoyl-sn-glycero-3-phosphocholine (POPC) to 1-palmitoyl-2- oleoyl-sn-glycero-3-phosphoglycerol (POPG) to cholesterol [wt: wt: wt] with 300 mM KCl (*SI Appendix*, Fig. S1 *A* and *B*). Following our work on Eag (20), valinomycin was added to the vesicles and the extravesicular solution was exchanged to 300 mM NaCl using a buffer-exchange column (Fig. 1*B*). The valinomycin-mediated K^+^-efflux generates a membrane voltage with an upper limit of about −145 mV, such that the inside is negative with respect to the outside. These polarized vesicles were immediately applied to a holey carbon grid and frozen for cryogenic electron microscopy (cryo-EM) analysis (Fig. 1*C* and *SI Appendix*, Fig. S1*C*). Grids for an unpolarized control lacking the buffer exchange step (i.e., with symmetric 300 mM KCl) were also prepared.

The permeability of these KCNQ1-containing liposomes to small ions was tested using a liposome flux assay (Fig. 1*D*) (44). The vesicles prepared in 300 mM KCl (without valinomycin) were diluted into a buffer with a fluorescent dye, 9-amino-6-chloro-2-methoxyacridine (ACMA), and isotonic NaCl to generate a K^+^ gradient. The proton ionophore carbonyl cyanide m-chlorophenylhydrazone (CCCP) was added to allow H^+^ influx, which leads to quenched ACMA fluorescence. Without valinomycin, no flux was detected, consistent with the channel being tightly closed under these conditions. Subsequent addition of the K^+^-selective ionophore valinomycin gave rise to rapid quenching of ACMA in both KCNQ1 proteoliposomes and in control liposomes without protein (Fig. 1*D*), indicating that the valinomycin-generated membrane potential is stable for at least a few minutes.

### Identification of Three Structural Classes in the Polarized Dataset.

We collected large cryo-EM datasets on polarized and unpolarized vesicles using the same microscope, and the structures of KCNQ1 in both were determined using single-particle analysis (*SI Appendix*, Figs. S2–S4 and Table S1). As we found for the K_v_ channel Eag, channels were reconstituted exclusively in an inside-in orientation and thus, when polarized, experience hyperpolarizing (i.e., negative inside) potentials under the applied electric field (Fig. 1*C*). After two rounds of three-dimensional (3D) classification to select for the best subset of particles in each dataset, we carried out 3D classification without alignment with a mask on the TM domain while imposing C4 symmetry (*SI Appendix*, Fig. S2). The unpolarized dataset showed little heterogeneity: 88% of the particles were in a homogeneously “up” (detailed below) conformation that closely resembled the detergent structure of KCNQ1, while the remaining particles were in an indeterminate state. Meanwhile, the polarized dataset was noticeably more heterogeneous, with only 34% of particles in a homogeneously up conformation, 19% consistent with an “intermediate” conformation, 10% consistent with a “down” conformation, and the remaining indeterminate. Classification without symmetry on a symmetry-expanded particle set showed that these 34% of particles had all four voltage sensors in an up conformation—suggesting that these channels are in vesicles that have lost the ion gradient, and likely do not reflect the distribution of voltage sensor states under the applied potential. Similar classification on the remaining 66% of particles showed classes with voltage sensors in different states, indicating that the higher proportion of indeterminate particles in the polarized dataset is due to a mixture of conformations. In summary, the observation of distinct structural classes for the voltage sensor in the polarized but not the unpolarized dataset indicates that these conformational changes are likely caused by the application of an electric field (the alternative being due to Na^+^ in the external solution).

**Fig. 2.**
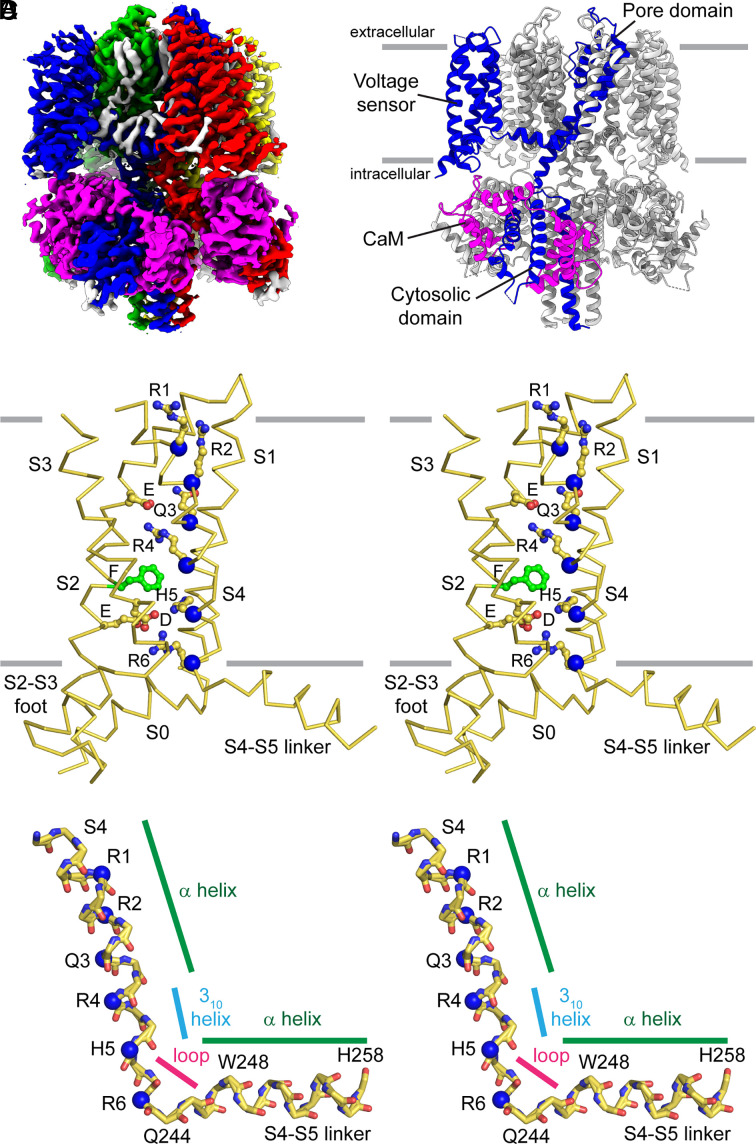
Structure of KCNQ1 in lipid vesicles with the voltage sensor in the up conformation. (*A*) Cryo-EM density map of the up structure of the KCNQ1 channel from the unpolarized dataset. Each channel subunit is shown in a different color and calmodulin (CaM) is shown in magenta. Bound lipids or sterols are shown as gray density. (*B*) Structure of the KCNQ1–CaM complex (cartoon representation) showing domains within one monomer (blue) from the N- to C-terminus: voltage sensor, pore domain, and the cytosolic domain. The other monomers are colored gray for clarity and the bound calmodulin is colored magenta. (*C*) Stereoview of the KCNQ1 voltage sensor (Cα trace) in the up (depolarized) conformation. The six positive charges in S4 (α carbon marked by blue spheres), three negative charges in S2 and S3 (E160, E170, and D202), and the hydrophobic Phe in S2 (F167, green sticks) are shown in stick-and-ball representation. (*D*) Stereoview of the main chain in S4 and the S4–S5 linker (stick representation) in the up conformation. The α carbons of the six positive charges in S4 are marked by blue spheres. Regions with different secondary structures are indicated: α-helix (green), 3_10_ helix (cyan), and loop (magenta).

From the unpolarized dataset, the best up structure (C4-symmetric; *SI Appendix*, Fig. S3) had an overall resolution of 2.9 Å (Fig. 2*A* and *SI Appendix*, Fig. S4). We solved three structures from the polarized dataset (*SI Appendix*, Figs. S3 and S4): C4-symmetric up and intermediate structures with overall resolutions of 3.4 Å and 6.2 Å, respectively, and a C1-symmetric down structure from a symmetry-expanded particle set with an overall resolution of 6.8 Å. The up structures from the unpolarized and polarized datasets are nearly identical (*SI Appendix*, Fig. S5 *A* and *B*), so we focus on the better-resolved former structure. We note that one interesting difference between the two up structures regards the occupancy of K^+^ ions in the selectivity filter (*SI Appendix*, Fig. S5 *C* and *D*). In the polarized sample, due to the low extravesicular concentration of K^+^, density is only visible at the first and third positions in the selectivity filter, while density is present at all four positions in the unpolarized sample. Similar differences were observed in our previous study on Eag (20) and are qualitatively consistent with crystal structures of KcsA solved under symmetrical high and low K^+^ concentrations (45).

### The Up Conformation of the Voltage Sensor.

The up map is best defined in the TM domain, with local resolution estimates of ~2.4 to 2.8 Å for much of S1 through S6 (*SI Appendix*, Fig. S4*D*). Density for individual hydrogen-bonded water molecules is visible in the voltage sensor (*SI Appendix*, Fig. S6*A*). These water molecules do not represent a bulk water-filled crevice, but nevertheless undoubtedly contribute to the stabilization of positive-charged residues (20). Tightly bound phospholipid and sterol molecules (*SI Appendix*, Fig. S6*B*) are also visible at both the outer and inner leaflets of the membrane. These features are not discussed further in this paper but are highlighted to demonstrate the feasibility of obtaining high-quality cryo-EM reconstructions in lipid bilayers.

**Fig. 3.**
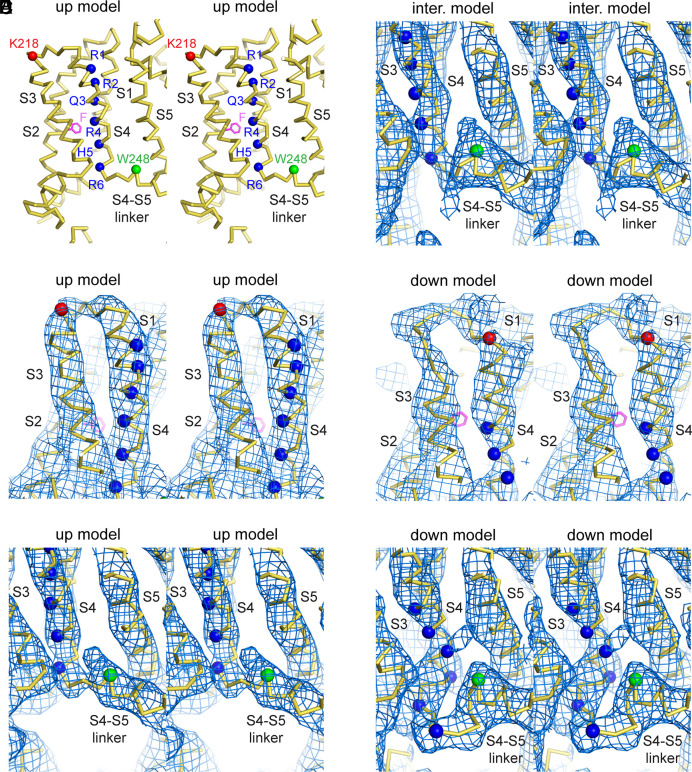
Characterization of electric field–induced movements in the KCNQ1 voltage sensor. (*A*) Stereoview (Cα trace) of the voltage sensor in the up model. For reference, the positions of the alpha carbons of the six positive charges in S4 are marked by blue spheres, that of K218 at the top of S3 is marked by a red sphere, and that of W248 at the end of the S4–S5 linker is marked by a green sphere. F167 in S2, which is part of the gating charge transfer center, is shown in magenta stick representation. (*B* and *E*) Stereoviews of the top part of S3 and S4 in the lowpass-filtered up model and map (unpolarized dataset, *B*) and in the down model and map (polarized dataset, *E*). (**C*, *D* and *F**) Stereoview of the bottom part of S4 and the S4–S5 linker in the lowpass-filtered up model and map (unpolarized dataset, *C*), intermediate (inter.) model and map (polarized dataset, *D*), and in the down model and map (polarized dataset, *F*). The up map was lowpass filtered to 6.5 Å to facilitate comparison to the down and intermediate maps.

A structural model was built by fitting the detergent structure of KCNQ1 (40) and making adjustments where needed (Fig. 2 *B*–*D*). The up structure in lipid bilayers is very similar to the depolarized structure in detergent micelles. The S4–S5 linker is an α-helix from I257 to G245 and a short loop (Q244 to D242) connects the S4–S5 linker to S4 (Fig. 2*D*). The S4 is a 3_10_ helix from V241 to R237 and an α-helix from L236 to T224 (Fig. 2*D*). The S3–S4 loop is partially flexible—with the four residues (GQVF) in between K218 (top of S3) and A223 (top of S4) not well defined—a point we shall return to later. The six positive-charged residues in S4 are positioned as such (Fig. 2*C*): R6 (R243) lies below the gating charge transfer center. H5 (H240) occupies the gating charge transfer center consisting of F167 from S2 and the negative-charged E170 and D202 from S2 and S3, respectively. R4 (R237) is directly above the gating charge transfer center and interacts with E160 in S2. Q3 (Q234), R2 (R231), and R1 (R228) lie further toward the extracellular side of the membrane. Q3 lies within the voltage sensor helical bundle, R2 is at the periphery, and R1 is pointed toward the headgroups of the phospholipid bilayer (Figs. 2*C* and 3 *A*–*C*).

### The Down and Intermediate Conformations of the Voltage Sensor.

The intermediate and down maps are less well defined due to heterogeneity, but clear differences in the main chain compared to the up map (modeled in Fig. 3*A*) were used to build partial models. We compare the down (Fig. 3 *E* and *F*) and intermediate (Fig. 3*D*) maps to an up map that is filtered to a comparable resolution (Fig. 3 *B* and *C*). Compared to the up map, the down map shows a dramatic change in the bottom half of S4, near the intracellular surface (Fig. 3 *C* and *F* and Movie S1). At the intracellular surface, the loop connecting S4 to the S4–S5 linker helix becomes lengthened by eight or nine amino acids. The lengthening occurs while the S4–S5 linker helix on the C-terminal side of W248, whose side chain density is apparent even at the lower resolution of the down map, remains unchanged in its position. Given that the S4–S5 linker helix does not move, the lengthened loop must result from amino acids originating in the downward displacement of the S4 helix and the four residues in the S3–S4 loop. The density in the newly formed extended loop suggests that as S4 moves downward, it forms a broken helix ~30° relative to the bilayer normal, and an extended loop (Fig. 3*F* and Movie S1). On the extracellular side, the top of helical densities for both S3 and S4 appears embedded about one turn below the expected plane of the extracellular membrane surface, while a short loop connecting them reaches to the extracellular surface (Fig. 3 *B* and *E*).

In the absence of side chain density, given the large conformational change in the position of S4 required to form the large loop on the intracellular side, we could not build a model of this region with certainty in the polypeptide register. We built two tentative polyalanine models into the continuous main chain density, one invoking a three helical turn displacement of S4 and the other invoking a two helical turn displacement (*SI Appendix*, Fig. S7). One and four helical turn models are incompatible with the observed density. The three helical turn displacement, which would place Q3-R6 below, R2 in, and R1 above the gating charge transfer, more reliably accounts for density, but additional data will be needed to establish this conclusion. We note at this point that the mechanism presented in the current study (to be discussed) does not rely on modeling side chains or the detailed register of the S4 helix, because the main chain movements we do observe clearly interfere with the PIP_2_-binding site and thus explain the basic mechanism of this channel’s gating.

In contrast to the down structure, the intermediate structure largely preserves the secondary structure of the up conformation but displays a ~4 Å downward displacement of the loop connecting S4 to the S4–S5 linker (Fig. 3 *C* and *D*). As in the down structure, the S4–S5 linker helix does not move appreciably. Given that the motion is likely to be a rigid body movement of S4, we included S4 sidechains in the structural model of the intermediate conformation. The intermediate structure places R6 and H5 below the gating charge transfer center; R4 in the gating charge transfer center; and Q3, R2, and R1 above the gating charge transfer center.

In all three structures, the pore appears tightly closed, as expected in the absence of PIP_2_ (*SI Appendix*, Fig. S8*C*). The pore radius is ~1 Å at S349 in the up structure, which is notably smaller than the radius of a hydrated K+ ion (~4 Å). While the side chain of S349 is not visible in the intermediate and down maps, the position of S6 is the same as in the up structure with a closed pore and different from the PIP_2_-bound structure with an open pore (*SI Appendix*, Fig. S8*C*), thus being consistent with a closed pore.

## Discussion

### The Relationship between Voltage Sensor Movements and PIP_2_ Binding.

**Fig. 4.**
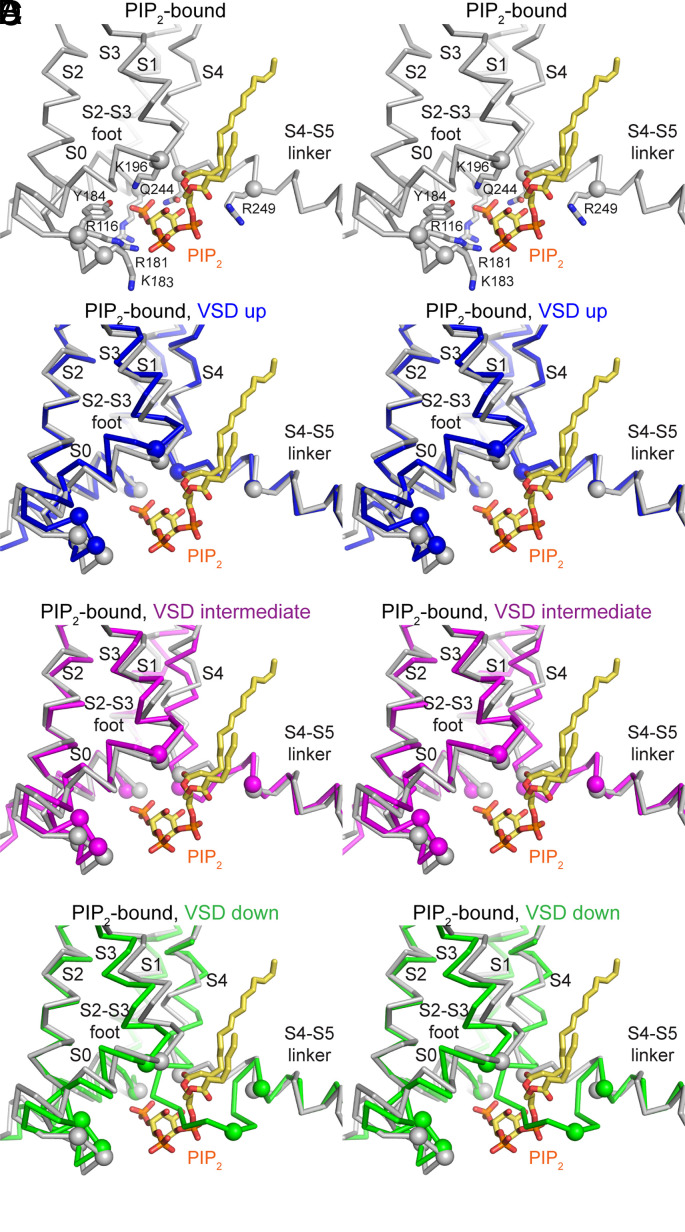
The relationship between voltage sensor movements and PIP_2_ binding. (*A*) Stereoview (gray Cα trace) of the PIP_2_-bound structure of KCNQ1 (PDB ID: 6V01) (40) with the voltage sensor in the up conformation and an open pore. Positive-charged and polar residues that interact with PIP_2_ are labeled and shown as gray sticks (α carbon marked by gray spheres) and PIP_2_ is shown as yellow sticks. (*B*) Stereoview of the PIP_2_-free structure of KCNQ1 with the voltage sensor in the up conformation and a closed pore (blue Cα trace) and the PIP_2_-bound structure shown in panel *A* (gray Cα trace). (*C*) Stereoview of the PIP_2_-free structure of KCNQ1 with the voltage sensor in the intermediate conformation and a closed pore (magenta Cα trace) and the PIP_2_-bound structure shown in panel *A* (gray Cα trace). (*D*) Stereoview of the PIP_2_-free structure of KCNQ1 with the voltage sensor in the down conformation and a closed pore (green Cα trace) and the PIP_2_-bound structure shown in panel *A* (gray Cα trace). In panels (*B*–*D*), the α carbon positions of the PIP_2_-interacting residues are shown as spheres in the same color as the α carbon trace.

The three structures presented in this study delineate the movement of the S4 helix in the KCNQ1 channel in response to polarization, while the pore of the channel is closed in all the three cases due to the absence of PIP_2_ in our preparation. The structure of PIP_2_-bound KCNQ1 with an open pore and the voltage sensor in the up conformation was already determined (40, 46, 47). By comparing these structures, we can deduce how the voltage sensor movements relate to PIP_2_ binding. The PIP_2_-binding site in KCNQ1 comprises positive-charged and polar residues in the S4–S5 linker, S4 helix, the S2–S3 foot, and the S0 helix (Fig. 4*A*, see also Fig. 2*C*). This structure of the pocket is maintained when the pore of the channel is open or closed as long as the voltage sensor is in the up conformation (Fig. 4*B*). In other words, when the voltage sensor is up, PIP_2_ can bind to this pocket and promote channel opening, as described previously (33–36, 40). We note that all the structures of KCNQ1 solved in the presence of PIP_2_ show an open pore, but only some also show a large conformational change in the cytoplasmic domain (*SI Appendix*, Fig. S8 *C–E*) (40, 46). The relationship between the two is not clear, but it is apparent that PIP_2_-binding causes the pore to open, which is what we focus on here.

Overlays of the intermediate (Fig. 4*C*) and down (Fig. 4*D*) voltage sensor conformations with the PIP_2_-bound, voltage sensor up conformation show that the PIP_2_-binding site is reshaped when S4 moves. The position of S4 in the down conformation sterically occludes the PIP_2_-binding site altogether (Fig. 4*D*). Thus, while the voltage sensor is in the down conformation, PIP_2_ cannot bind to the channel and open the pore. In the intermediate conformation, the residues that bind PIP_2_ are displaced relative to one another due to the movement of S4 (Fig. 4*C*). This intermediate conformational change would likely alter the affinity of the PIP_2_-binding site, but it might not definitively preclude the binding of PIP_2_.

### Voltage-Dependent Regulation of PIP_2_ Binding in KCNQ1.

**Fig. 5.**
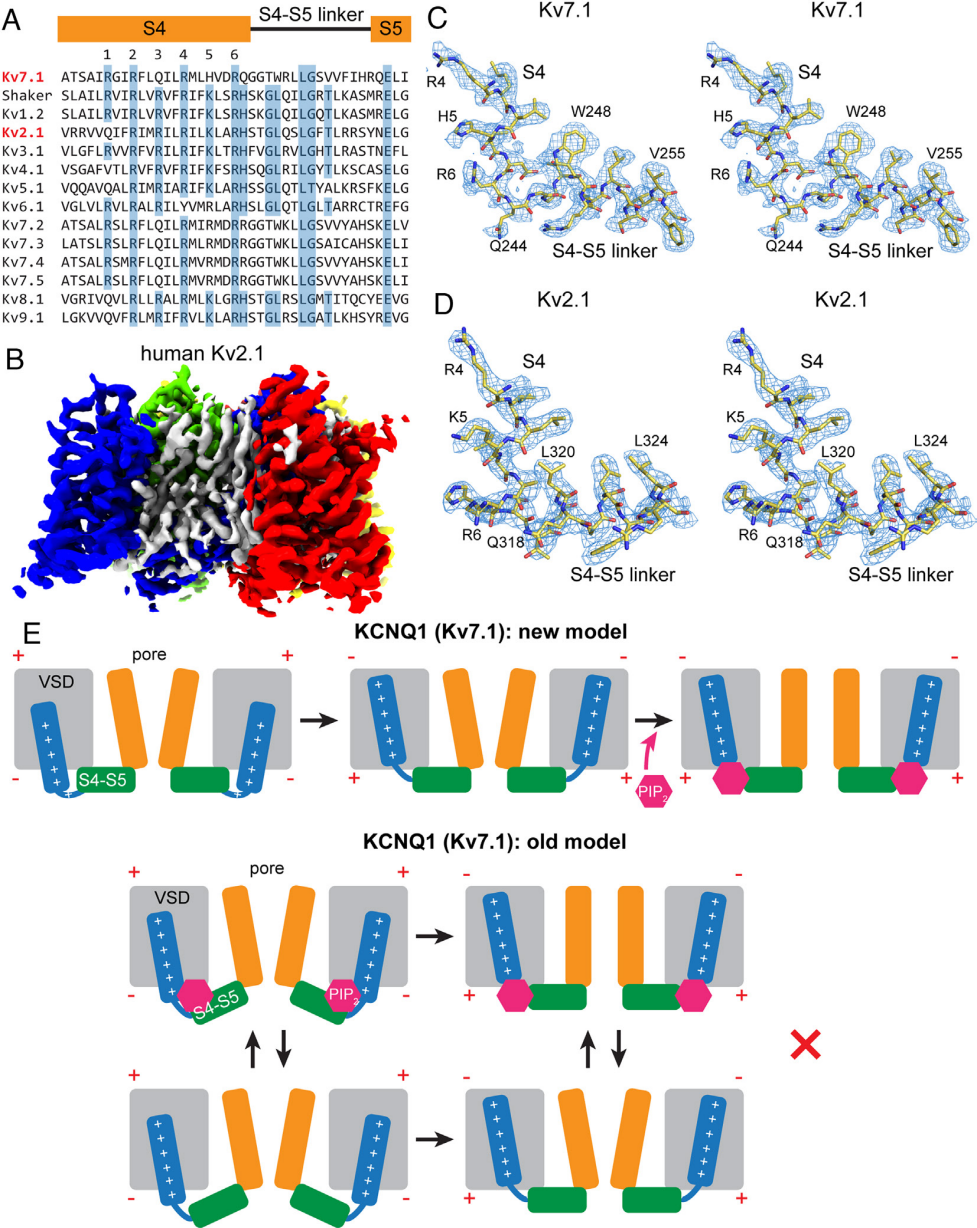
The structure of S4 and the S4–S5 linker of K_v_7.1 (KCNQ1) and K_v_2.1 determined in lipid vesicles. (*A*) Sequence alignment of S4 and the S4–S5 linker for all domain-swapped K_v_ channel families. All members of the K_v_7 family are included for comparison to K_v_7.1. Residues conserved across all families are highlighted in blue. (*B*) Cryo-EM density map of the human K_v_2.1 channel determined in lipid vesicles. Each channel subunit is shown in a different color and associated lipids or sterols are shown as gray density. (*C* and *D*) Stereoviews of the connection between S4 and the S4–S5 linker (stick representation) in the depolarized conformations of Kv7.1 (*C*) and Kv2.1 (*D*) overlaid with cryo-EM density (blue mesh). (*E*) Cartoons depicting the new gating model (*Top*) and the old gating model (*Bottom*) for KCNQ1. The pore domain is colored orange, the voltage sensor is gray, the S4 helix is blue, the S4–S5 linker is green, and PIP_2_ is depicted as a magenta hexagon. In the new model, the voltage sensor regulates the binding of PIP_2_ by occluding the binding site in the down conformation (*Left*). When membrane depolarization occurs, the voltage sensor moves to the up conformation (*Middle*), which then allows PIP_2_ to bind to the channel and open the pore (*Right*). In the old model that is inconsistent with our data, a PIP_2_-binding site is present in the down conformation, allowing PIP_2_ to bind to the channel.

A mechanism for voltage-dependent regulation of KCNQ1 channel activity thus follows (Fig. 5*E*). We have made a movie to visualize the sequence of events (Movie S2). At hyperpolarized membrane voltages (i.e., at the resting potential of a cell, corresponding to our polarized vesicles), the voltage sensor is in the down conformation, which prevents PIP_2_ from binding because the site is occluded. Depolarization drives the S4 helix up, which is coupled to the formation of the PIP_2_ binding site. PIP_2_ can then bind, which causes the pore to open through an allosteric mechanism (40, 46, 47). In other words, KCNQ1 activity is modulated by a ligand (PIP_2_), the binding of which is regulated by the voltage sensor. This is different in detail from a mechanism in which the binding of PIP_2_ permits voltage sensor conformational changes to regulate the pore through direct mechanical coupling (Fig. 5*E*).

This voltage-dependent regulation of PIP_2_-binding mechanism is compatible with electrophysiological studies of KCNQ channels, which show that voltage sensor movements slightly precede pore opening (48, 49), that PIP_2_ is required for activity (33–36), and that in the absence of PIP_2_, the voltage sensors move but the pore does not open (38). Moreover, if the voltage sensors were to perform work directly on the pore to open it, and if PIP_2_ was required for this coupling, one would expect a shift in the voltage activation midpoint (i.e., as measured by the movement of S4) depending on whether PIP_2_ is bound or not. But, the movement of S4 happens at the same membrane voltage whether PIP_2_ is present in the membrane or not (38), suggesting that the voltage sensors do not perform work on the pore at depolarized potentials to open it. Finally, voltage clamp fluorometry using a reporter on the S3–S4 linker shows two components: a larger fluorescence change that has a midpoint of ~−60 mV and a smaller change in fluorescence with a midpoint of ~30 mV (50, 51). The pore begins to open during the first (more negative voltage) fluorescence change, which has led to the proposal that there are two open states of the channel. These observations could be related to the intermediate and up voltage sensor conformations that we observe.

### Unique Features of the KCNQ1 Voltage Sensor.

KCNQ1 is unique among domain-swapped K_v_ channels in its requirement of PIP_2_ to open. To this point, a comparison of the KCNQ1 structure to that of a different domain-swapped K_v_ channel is informative. A primary sequence alignment from S4 through the S4–S5 linker for the Shaker channel, one member from each of the domain-swapped K_v_ channel families (K_v_1-9), and other members of the K_v_7 (KCNQ) family, is given in Fig. 5*A*. Stereoviews of the S4 to S4–S5 helix linker connection along with cryo-EM density are shown for the depolarized structures of K_v_7.1 (Fig. 5*C*, see also Fig. 2*D*) and human K_v_2.1 (52), both in lipid vesicles (Fig. 5*D*). The K_v_2.1 structure was determined to an overall resolution of 3.0 Å (Fig. 5*B* and *SI Appendix*, Fig. S9). An important difference between K_v_7.1 and K_v_2.1 becomes apparent at the junction of S4 and the S4–S5 linker. In KCNQ1, these residues form a helix–loop–helix motif, with three flexible amino acids (G245, G246, and T247) in or adjacent to the loop. Meanwhile, in K_v_2.1, the junction is a helix–turn–helix motif. In other words, K_v_7.1 has a natural propensity to form a loop in this region, which is not shared by the domain-swapped channel K_v_2.1. The S4 movement that we observe in the intermediate and down conformations is centered exactly at this flexible “GGT” motif. Moreover, this motif (and in fact, most of the S4–S5 linker) is conserved among K_v_7 family members but is absent in other domain-swapped K_v_ channels (Fig. 5*A*), indicating that it is a hallmark of the PIP_2_-gated KCNQ channels. Whether Shaker-related channels under an applied electric field undergo similar or distinct voltage sensor movements compared to KCNQ1 remains to be seen.

### Implications for Other Voltage-Dependent Channels.

In KCNQ1, membrane polarization causes the S4 helix to displace by one helical turn (~5 Å) in the intermediate structure and most likely three helical turns (~15 Å) in the down structure, but the S4–S5 linker helix does not move appreciably (*SI Appendix*, Fig. S8*A*). One might argue that this is because the pore is closed due to the lack of PIP_2_. But, structures of KCNQ1 with an open pore are known (40, 46), and the S4–S5 linker helix occupies a similar position in those as well (*SI Appendix*, Fig. S8*B*). This finding suggests that the position of the S4–S5 linker helix is not strictly coupled to pore opening and closing in the KCNQ1 channel. It is still possible that small movements in the S4–S5 linker bias the conformational state of the pore, but S4–S5 helix movements are minimal when the S4 helix is displaced.

What does this static S4–S5 helix in KCNQ1, if anything, suggest for other domain-swapped channels like Shaker-related K_v_ channels, Na_v_, or Ca_v_ channels? When the first molecular structure of a eukaryotic voltage-dependent ion channel—the Shaker-related K_v_1.2—was determined (16), the S4–S5 linker was found to contact S6 directly. A simple mechanical model for voltage-dependent regulation of the pore was proposed: When S4 moves in response to an electric field, the amino terminal end of the S4–S5 linker is displaced, applying a force on S6 and causing pore closure through straightening of the S6 helix at a conserved “PxP” motif (53). Many years later, structures of chemically cross-linked or trapped Na_v_ channels (21, 24, 25) and metal bridge–linked K_v_4.2 channels (26) showed that it is indeed possible to trap channels in conformations consistent with the simple mechanical model. We observe in the present study, however, that KCNQ1 does not function according to this model. While KCNQ1 is an outlier among domain-swapped voltage-dependent channels for the reasons discussed above, we remain open minded to the possibility that the simple mechanical model assumed for other domain-swapped voltage-dependent ion channels (16, 21, 24, 25), despite support from mutational and chemical crossbridge data, could be incorrect. Mutations and chemical crossbridges likely do not replicate the forces applied to a polarized voltage sensor in membranes because an electric force field acts on all charged atoms spread throughout the protein. Ultimately, to know whether the simple mechanical model is correct for other domain-swapped channels, we will need to determine their structures in lipid bilayers with an applied electrostatic force field.

### Comparison of Voltage Sensor Movements in EAG and KCNQ1.

**Fig. 6.**
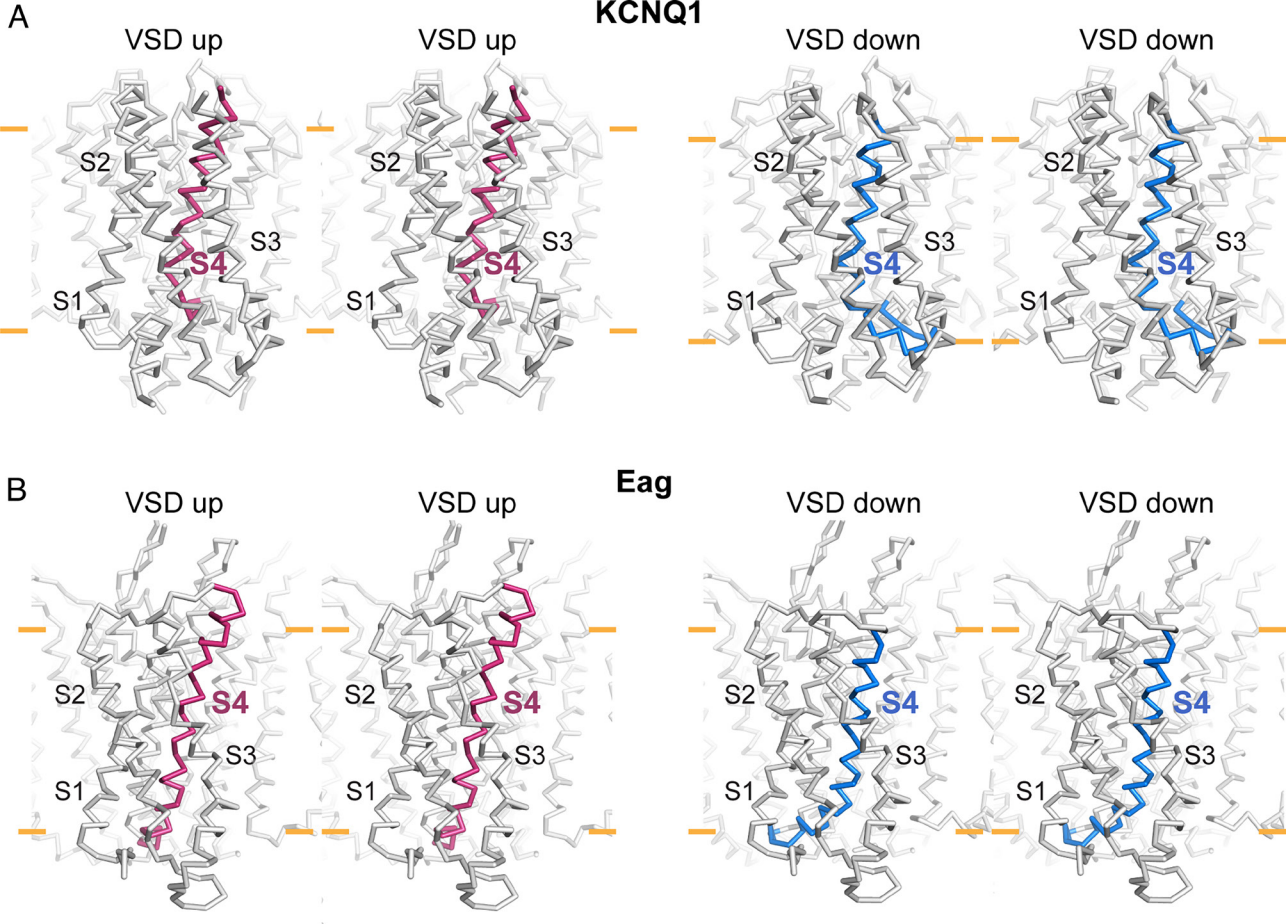
Comparison of voltage sensor movements in KCNQ1 and Eag. (*A* and *B*) Stereoviews of KCNQ1 (*A*) and Eag (*B*) showing the up conformation (*Left*, red S4) and the down conformation (*Right*, blue S4). The view looks through one voltage sensor in each channel toward the pore axis. The approximate position of the lipid membrane bilayer is marked by yellow lines and the protein is shown in Cα trace representation.

We now know how the voltage sensors in two potassium channels, KCNQ1 (domain-swapped) and Eag (nondomain-swapped) (20), undergo conformational changes in response to an applied voltage difference across the membrane. For comparison, side views of the voltage sensors in these channels are shown in Fig. 6. In both channels, as S4 displaces downward (i.e., toward the cytoplasm), an extended interfacial segment is formed through a break in S4, which is accompanied by a remodeling of the connection between S4 and the S4–S5 linker helix (KCNQ1; Fig. 6*A*) or S4 and S5 (Eag; Fig. 6*B*) (20). Apparently, because S4 is both charged and hydrophobic, an interfacial location is energetically more favorable than an aqueous location. The extra amino acids that account for the downward displacement of S4 originate from the S3–S4 linker and the top of S3 in both channels.

While the S4 displacement and interfacial helix formation are similar in KCNQ1 and Eag, the helices bend in opposite directions with respect to the pore. In Eag, the polarized S4 bends toward the pore axis, causing it to clamp down on the pore-lining S6 helix, which prevents pore opening (Fig. 6*B*) (20). In KCNQ1, the polarized S4 bends away from the pore axis so that it occludes the PIP_2_-binding site. These variations show how the same structural element—a voltage sensor—confers conformational sensitivity to an electric field in two K_v_ channels that differ both in their voltage sensor configuration (i.e., domain-swapped versus nondomain-swapped) and in their modulation by other effectors. Future studies of other voltage-dependent ion channels might uncover other interesting mechanisms for coupling the movement of S4 to gating the pore.

### On the Magnitude of S4 Displacement in KCNQ1.

As we state above, our inability to define the register of the S4 helix main chain (*SI Appendix*, Fig. S7) prevents us from distinguishing with certainty whether the down map, with its occluded PIP_2_-binding site, corresponds to a two or three helical turn displacement of S4. A two-turn displacement was anticipated because that is what we observed in a polarized Eag channel (20), and what has been seen in cross-linked Na_v_ and HCN voltage sensors (21, 23–25). Moreover, if S4 can displace three helical turns, it must pass through a two-turn-displaced intermediate. Why then would we not observe this intermediate? A possible answer lies in the unique S4 sequence of KCNQ1, which contains a neutral glutamine at “charged” position 3 (Fig. 5*A*). A two helical turn displacement would place the neutral glutamine into the highly negative-charged gating charge transfer center (Fig. 2*C*). For this reason, conformations with one or three helical turn displacements (which both place an arginine in the gating charge transfer center) may be energetically more stable in KCNQ1 than a conformation with two. If this is the case, a two helical turn displacement would function as a transient energy barrier in the conformational change of the voltage sensor.

The notion that different residues are stable to varying degrees when they occupy the gating charge transfer center is apparent from functional measurements in other voltage-dependent ion channels. For instance, in the Shaker channel, it has been shown that it is more favorable for a lysine than an arginine to occupy the gating charge transfer center (8). Depending on the position of the substitution within the S4 helix, either the open or the closed state of the channel can be stabilized (corresponding to an up or down conformation of the voltage sensor). Given that even two positive-charged residues, arginine or lysine, can differ in their relative stability, it seems quite possible that a neutral glutamine behaves differently than an arginine.

It is also useful to look at another example: Consider the Shaker channel and the domain-swapped channel K_v_2.1 (Fig. 5*A*). Both have lysine at the fifth position (K5) in the gating charge transfer center. In Shaker, all four residues above the gating charge transfer center are arginines, while K_v_2.1 has a glutamine at position one (Q1) followed by three arginines. The gating charge estimated by nonlinear membrane capacitance for Shaker is ~12 to 14 elementary charges per channel (~3 to 3.5 per voltage sensor) and that for K_v_2.1 is only ~6 to 7 per channel (~1.5 to 2 per voltage sensor) (54, 55). The gating charge estimates for Shaker indicate that the first residue (R1) does not traverse the membrane potential. Yet the presence of a neutral glutamine at the first position in K_v_2.1 reduces the apparent gating charge in half. We suppose that in the down conformation of K_v_2.1, there is a tendency for R2 to neutralize the extracellular negative-charged residue while R3 occupies the gating charge transfer center, consistent with the net movement of ~2 gating charges. These observations are consistent with the idea that it is more favorable for an arginine (compared to a glutamine) to interact with negative-charged residues in the voltage sensor.

In summary, this study provides the structural description of a domain-swapped K_v_ channel in a lipid bilayer under the influence of a polarizing electric field. The structures reveal a mechanism in which the voltage sensor regulates the affinity of PIP_2_, thus controlling its ability to gate the pore.

## Materials and Methods

### Cell Lines.

Sf9 (*Spodoptera frugiperda* Sf21) cells were used for production of baculovirus and were cultured in Sf-900 II SFM medium (GIBCO) supplemented with 100 U/mL penicillin and 100 U/mL streptomycin at 27 °C under atmospheric CO_2_.

HEK293S GnTl^−^ cells were used for protein expression and were cultured in Freestyle 293 medium (GIBCO) supplemented with 2% fetal bovine serum, 100 U/mL penicillin, and 100 U/mL streptomycin at 37 °C in 8% CO_2_.

### Expression and Purification of the KCNQ1–Calmodulin Complex.

The KCNQ1(K_v_7.1)–calmodulin complex (hereby referred to as KCNQ1) was expressed and purified as described before (40), with slight modifications. We used a construct corresponding to human KCNQ1 with N-terminal and C-terminal truncations, leaving residues 76 to 620. The construct was cloned into the BacMan expression vector with a C-terminal green fluorescent protein (GFP)-His_6_ tag linked by a preScission protease (PPX) site (56). A separate BacMan expression vector without a tag was used for vertebrate calmodulin (CaM).

Bacmids were generated for KCNQ1 and CaM using DH10Bac *Escherichia coli* cells. Baculoviruses for KCNQ1 and CaM were produced in SF9 cells transfected with bacmid DNA using the Cellfectin II reagent (Invitrogen). Baculovirus was amplified three times in suspension cultures of SF9 cells grown at 27 °C. Four liters of suspension cultures of HEK293S GnTI^-^ at ~3 × 10^6^ cells/mL were infected with 12% (v/v) of 5:1 KCNQ1:CaM baculovirus at 37 °C for ~8 h. Protein expression was induced by adding 10 µM sodium butyrate, and the incubation temperature was changed to 30 °C for the duration of expression. Cell pellets were harvested ~48 h after induction and flash frozen in liquid nitrogen for later use.

Four liters of cell pellet were resuspended in ~100 mL of lysis buffer (25 mM Tris pH 8.0, 300 mM KCl, 1 mM MgCl_2_, 5 mM CaCl_2_, 2 mM dithiothreitol (DTT), 1 µg/mL leupeptin, 1 µg/mL pepstatin, 1 mM benzamidine, 1 µg/mL aprotinin, 1 mM phenylmethylsulfonyl fluoride, 1 mM 4-(2-aminoethyl) benzenesulfonyl fluoride, and 0.1 mg/mL DNase), stirred for 10 min at 4 °C, and Dounce homogenized with a loose pestle till homogenous. The resultant suspension was clarified by centrifugation at 39,800 × g for 15 min at 4 °C. The pellet was resuspended in ~100 mL of lysis buffer and Dounce homogenized with a tight pestle. To extract the KCNQ1–CaM complex, we added 15 mL of a 10%:2% n-nodecyl-β-D-maltopyranoside (DDM):cholesteryl hemisuccinate (CHS) mixture and stirred for 1 h at 4 °C.

The mixture was clarified by centrifugation at 39,800 × g for 30 min at 4 °C. The supernatant was bound to ~2.5 mL GFP nanobody-coupled Sepharose resin (prepared in-house) (57) in a 250-mL conical centrifuge tube (Corning) by gentle rotation for 1 h at 4 °C. The resin was transferred to a glass gravity flow column (Bio-Rad) and washed with ~30 column volumes of wash buffer (10 mM Tris pH 8.0, 300 mM KCl, 0.05%:0.01% DDM:CHS, 1 mM CaCl_2_, and 2 mM DTT). The resin was resuspended in five column volumes of wash buffer, PPX (prepared in-house) was added at a concentration of 0.05 mg/mL to remove the GFP tag, and the solution was rotated for 1 h at 4 °C. The cleaved protein was collected in the flow through and a subsequent wash step with five column volumes of wash buffer. The protein was concentrated to ~500 µL at 3,000 × g and 4 °C using a 15-mL Amicon spin concentrator with a 100-kDa molecular weight cutoff membrane. The concentrated protein was filtered through a Corning 0.2 µm spin filter and then purified by size-exclusion chromatography (SEC) using a Superose 6 Increase column (10/300 GL) preequilibrated with SEC buffer (10 mM Tris pH 8.0, 300 mM KCl, 0.025%:0.005% DDM:CHS, 1 mM CaCl_2_, and 5 mM DTT). Fractions containing hKCNQ1 and calmodulin (*SI Appendix*, Fig. S1 *A* and *B*) were pooled and concentrated to an A_280_ of 3.8 mg/mL at 3,000 × g and 4 °C using a 4-mL Amicon spin concentrator with a 100-kDa molecular weight cutoff membrane. Purified protein was immediately used for reconstitution into liposomes.

### Reconstitution of the KCNQ1–Calmodulin Complex and Cryo-EM Grid Preparation.

The purified KCNQ1 complex was reconstituted into liposomes consisting of 90%: 5%: 5% POPC:POPG:cholesterol (wt: wt: wt, Avanti Polar Lipids) (20). The phospholipids and sterol were mixed together in chloroform at a concentration of 10 mg/mL. Ten milligrams of the lipid mixture were dried to a thin film in a screw-top glass tube under a gentle stream of argon. The lipid film was further dried for ~3 h in a room-temperature vacuum desiccator, and then resuspended at a concentration of 10 mg/mL by gentle vortexing in reconstitution buffer (10 mM Tris pH 8.0, 300 mM KCl and 1 mM DTT). Small unilamellar vesicles (SUVs) were formed by bath sonication (Branson Ultrasonics M1800) at room temperature till the solution was mostly transparent (A_400_ ~ 0.2), which typically took ~40 min. To permeabilize but not solubilize the lipid vesicles, the detergent C_12_E_10_ was added to the 10 mg/mL lipid stock solution to a final concentration of 2 mg/mL (5:1 lipid:detergent, wt/wt) and incubated on ice for ~15 min. Two hundred microliters of this permeabilized vesicle solution was mixed with 27 µL of the purified KCNQ1 complex (3.8 mg/mL) and 173 µL of reconstitution buffer, giving a total reaction volume of 400 µL (chosen to ensure proper mixing in a 1.5 mL Eppendorf tube), a protein:lipid ratio of 1:20 (wt/wt), and a final lipid concentration of 5 mg/mL. The lipid–protein–detergent mixture was incubated on ice for ~1.5 h. Detergent was removed using adsorbent Bio-Beads SM-2 resin (Bio-Rad) by adding 20 mg of a 50% (wt/vol) Bio-Beads slurry in reconstitution buffer and rotating at 4 °C for ~14 h. The biobeads procedure was repeated twice again for 3 h each at 4 °C to ensure complete removal of detergent. The suspension was bath sonicated briefly (twice for 10 s each) after the biobeads step to minimize vesicle clumping.

Polarized and unpolarized vesicles were prepared from the same batch of proteoliposomes. From an 8 mM stock in dimethyl sulfoxide, 2 µM valinomycin was added to the proteoliposomes and incubated for 30 min on ice. Polarized vesicles were prepared as follows: 70 µL of the above solution was added to a 0.5-mL Zeba spin desalting column (40 kDa cutoff, Thermo Scientific), preequilibrated with sodium reconstitution buffer (10 mM Tris pH 8.0 and 300 mM NaCl), to exchange the external K^+^ for Na^+^. The sample was centrifuged for ~20 to 30 s at room temperature at 1,500 × g and ~20 µL of flow-through containing vesicles was collected. The residual external K^+^ concentration is about 1 mM (20). Onto a glow-discharged Quantifoil R1.2/1.3 400 mesh Au grid, 3.5 µL of the polarized vesicle solution was immediately applied. The vesicle solution was incubated on the grid for 3 min at 20 °C under a humidity of 100%. The grid was then manually blotted from the edge of the grid using a piece of filter paper. Another 3.5 µL of the polarized vesicle solution was applied to the same grid for 20 s (58), and then the grid was blotted for 3 s with a blotting force of 0 and flash frozen in liquid ethane using a FEI Vitrobot Mark IV (FEI). Each grid with polarized vesicles used a freshly buffer exchanged sample. Grids for the unpolarized vesicles were frozen by skipping the buffer exchange step, i.e., directly applying the proteoliposomes (with valinomycin) on the Quantifoil grids.

### Expression and Purification of K_v_2.1.

Full-length human K_v_2.1 (NP_004966.1) with a C-terminal GFP-His_6_ tag linked by a PPX site and full-length 14-3-3 protein epsilon (empirically found to increase the yield of K_v_2.1, XP_040497056.1) were both cloned into a pBig1a vector from the biGBac system (59). Bacmids and baculovirus were generated, and protein was expressed in HEK293S GnTI^-^ cells as described above for KCNQ1.

The channel (hK_v_2.1) was purified following essentially the same protocol as KCNQ1 except that 150 mM KCl (instead of 300 mM KCl) was used for the wash buffer and calcium chloride was not included after the lysis buffer step. The final purification step entailed SEC on a Superose 6 Increase column (10/300 GL) preequilibrated with SEC buffer (10 mM Tris pH 8.0, 150 mM KCl, 0.03%:0.006% DDM:CHS, and 5 mM DTT). Fractions containing hK_v_2.1 (*SI Appendix*, Fig. S9*A*) were pooled and concentrated to an A_280_ of 1.4 mg/mL at 3,000 × g and 4 °C.

### Reconstitution of K_v_2.1 and Cryo-EM Grid Preparation.

Purified protein was reconstituted into liposomes of 90%:5%:5% POPC:POPG:cholesterol prepared in 150 mM KCl using a protein:lipid ratio of 1:20 (wt/wt), following the same protocol as for KCNQ1. A fourfold-molar excess of hanatoxin (compared to hK_v_2.1 monomers) isolated from Chilean rose tarantula (*Grammostola rosea*) venom (60) was incubated with the proteoliposomes before freezing grids, but the toxin was not visible in the cryo-EM reconstructions. Grids were frozen exactly as described for unpolarized vesicles containing KCNQ1 (but without added valinomycin).

### Liposome Flux Assay.

The flux assay was carried out as described before (44), with minor modifications. The proteoliposome vesicles or control vesicles without protein (subjected to a mock reconstitution) prepared in 300 mM KCl were diluted 10-fold in isotonic sodium buffer (10 mM Tris pH 8.0 and 300 mM NaCl) immediately prior to the assay. Six microliters of the diluted vesicle solution was mixed with 6 µL ACMA solution (10 mM Tris pH 8.0, 300 mM NaCl, and 5 mM ACMA) and 12 µL buffer (10 mM Tris pH 8.0 and 300 mM NaCl). ACMA fluorescence was recorded every 5 s (excitation wavelength = 410 nm, emission wavelength = 490 nm) using a 384-well plate (Grainger) on a fluorescence plate reader (Tecan Infinite M1000). After the ACMA fluorescence stabilized, 6 µL of CCCP solution (10 mM Tris pH 8.0, 300 mM NaCl, and 15 mM CCCP) was added. The resultant KCNQ1-dependent flux, or in this case, the lack thereof because of the absence of PIP_2_, was measured. At the end of the assay, 2 µL of a 1.2-µM valinomycin solution (in 10 mM Tris pH 8.0 and trace dimethyl sulfoxide) was added to initiate K^+^ efflux from all the vesicles and determine the minimum ACMA fluorescence. The fluorescence data for each run were normalized by the fluorescence value right before the addition of CCCP (i.e., at 90 s). The normalized data were averaged across five independent measurements, and the mean and SDs are reported.

### Cryo-EM Data Acquisition and Processing.

Data for the polarized and unpolarized KCNQ1 liposomes were collected on the same microscope—a 300-keV FEI Titan Krios2 microscope located at the HHMI Janelia Research Campus. The microscope was equipped with a Gatan Image Filter (GIF) BioQuantum energy filter and a Gatan K3 camera. A total of 33,057 movies (polarized sample) or 19,998 movies (unpolarized sample) were recorded on Quantifoil grids in superresolution mode using SerialEM (61). The movies were recorded with a physical pixel size of 0.839 Å (superresolution pixel size of 0.4195 Å) and a target defocus range of −1.0 to −2.0 µm. The total exposure time was ~2 s (fractionated into 50 frames) with a cumulative dose of ~60 e^−^/Å^2^.

The data-processing workflow is detailed in *SI Appendix*, Figs. S2 and S3, and followed the same strategy we previously reported for Eag (20). Data processing was carried out using cryoSPARC v3.3.1 (62) and RELION 4.0 (63). The superresolution movies were gain-normalized, binned by a factor of 2 with Fourier cropping, and corrected for full-frame and sample motion using the Patch motion correction tool (grid = 15 × 10). Contrast transfer function parameters were estimated from the motion-corrected micrographs using the Patch CTF estimation tool, which uses micrographs without dose weighting. All subsequent processing was performed on motion-corrected micrographs with dose weighting. Particle picking was initially carried out using the Blob picker. 2D classes with clear protein density were used to train a TOPAZ picking model (64), which was used to pick additional particles. Particles with clear protein density after 2D classification were pooled and duplicate picks were removed. An ab initio model was generated from 2D classes with clear secondary structure features, and 3D classification and refinement was carried out either in cryoSPARC or RELION as detailed in *SI Appendix*, Figs. S2 and S3.

Data for the hK_v_2.1 liposomes were collected on a 300-keV FEI Titan Krios microscope located at the HHMI Janelia Research Campus. The microscope was equipped with a spherical aberration corrector (Cs corrector), a GIF BioQuantum energy filter, and a Gatan K3 camera. A total of 17,007 movies were recorded on a single Quantifoil grid in superresolution mode using SerialEM. The movies were recorded with a physical pixel size of 0.844 Å (superresolution pixel size of 0.422 Å) and a target defocus range of −1.0 to −2.0 µm. The total exposure time was ~2 s (fractionated into 50 frames) with a cumulative dose of ~60 e^−^/Å^2^. Data processing was carried out as described for KCNQ1 liposomes.

### Model Building and Refinement.

A structural model for the up conformation was built by docking the structure of KCNQ1–CaM in detergent micelles (PDB ID: 6UZZ) (40) into the up map and making adjustments where needed. The model was edited and refined using the ISOLDE (65) plugin in ChimeraX v1.2.0 (66) or WinCoot v0.98.1 (67) followed by real-space refinement in Phenix (68). The down and intermediate models were built starting from the up model. The up model was initially fit in the intermediate or down maps as a rigid body using Phenix. The S4 helix and the surrounding regions were manually adjusted and then a final step of real-space refinement was carried out in Phenix. The quality of the final models was evaluated using the MolProbity plugin in Phenix (*SI Appendix*, Table S1). Graphical representations of models and cryo-EM density maps were prepared using PyMOL (69) and ChimeraX.

## Supplementary Material

Appendix 01 (PDF)

Movie S1. Comparison of cryo-EM density in the up and down structures. This movie shows side-by-side views of the lowpass filtered up structure (left) and the down structure (right). The protein is shown as a blue Cα trace with S4 highlighted in red. First the up structure is rotated and then the down structure to show the difference in density between the two maps.

Movie S2. Sequence of conformational changes occurring during channel gating. The movie shows a side view of the morph between three structures of KCNQ1: with the pore closed and voltage sensor down (this work), with the pore closed and voltage sensor up (this work), and with the pore open and voltage sensor up (ML277 bound, PDB ID: 7XNK). The protein is shown in Cα trace representation and PIP_2_ is shown in yellow stick representation. The S4 and S6 of one subunit are colored blue and green, respectively. At hyperpolarized membrane voltages (i.e. the resting potential of a cell), the voltage sensor is in the down conformation, which prevents PIP_2_ binding. Depolarization drives the voltage sensor up, which then allows PIP_2_ to bind and promote opening of the pore. The sequence then runs in reverse. Note that the pore open and voltage sensor up structure with PIP_2_ bound (PDB ID: 6V01) was not used for the morph because it contains KCNE3, which causes a rotation of the voltage sensor relative to the pore and distracts from the pore opening we wish to show.

## Data Availability

Cryo-EM density maps of the KCNQ1 channel with the voltage sensor in the up, intermediate, and down conformation have been deposited in the electron microscopy data bank under accession codes EMD-40508 (70), EMD-40509 (71), and EMD-40510 (72), respectively. Atomic coordinates of the KCNQ1 channel with the voltage sensor in the up, intermediate, and down conformation have been deposited in the protein data bank under accession codes 8SIK (73), 8SIM (74), and 8SIN (75), respectively.
