# Supplementary material for: The membrane electric field regulates the PIP2-binding site to gate the KCNQ1 channel
Source: Proc Natl Acad Sci U S A. 2023 May 16;120(21):e2301985120. doi: 10.1073/pnas.2301985120 (PMC10214144; doi:10.1073/pnas.2301985120)
Supplement: Supplementary file 1 — Appendix 01 (PDF) [file pnas.2301985120.sapp.pdf]

PNAS

Supporting Information for

The membrane electric field regulates the PIP_2-binding site to gate the KCNQ1 channel.

Venkata Shiva Mandala[a,b] and Roderick MacKinnon[a,b,1].

[a]Laboratory of Molecular Neurobiology and Biophysics, The Rockefeller University, New York, NY, 10065; and [b]HHMI, The Rockefeller University, New York, NY, 10065.

[1]Correspondence to: Roderick MacKinnon.

Email: mackinn@rockefeller.edu.

This PDF file includes:

Figures S1 to S9
Table S1
Legends for Movies S1 to S2

Other supporting materials for this manuscript include the following:

Movie S1
Movie S2

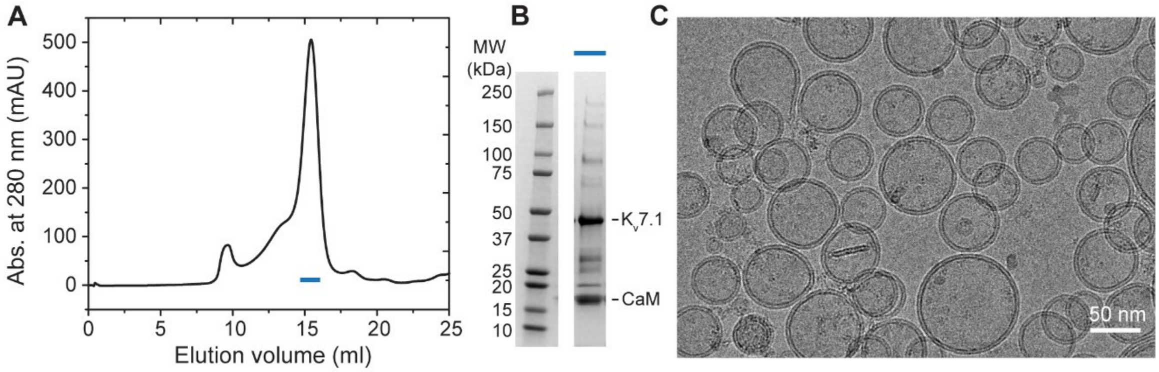

Figure S1. Purification of the KCNQ1-CaM complex and representative micrograph.

(**A**) Gel-filtration chromatogram of the KCNQ1 (K_v7.1) – calmodulin (CaM) complex on a Superose 6 Increase column. The fractions marked with a blue line (15-16 mL) were pooled and used for reconstitution. (**B**) SDS-PAGE gel of the selected fractions showing the presence of K_v7.1 and CaM. (**C**) Representative cryo-EM micrograph showing mostly unilamellar vesicles containing K_v7.1 (scale bar: 50 nm).

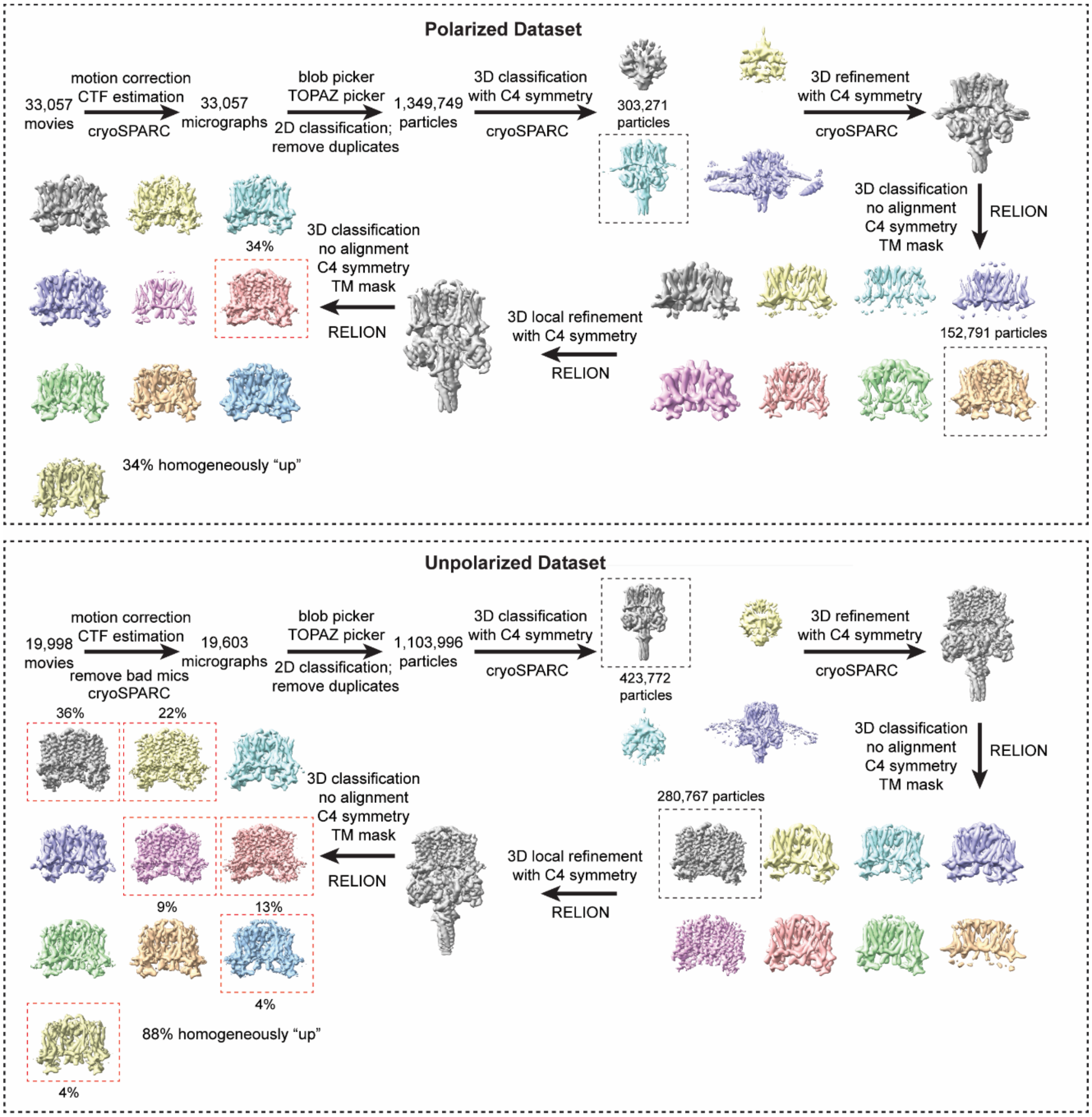

Figure S2. Three-dimensional classification of the polarized and unpolarized vesicle cryo-EM datasets.

Cryo-EM maps from refinement or classification are shown in solid surface. Classes selected for further refinement are marked by a dashed grey box. In the final step of classification, classes that were consistent with the “up” (depolarized) voltage sensor are marked by a dashed red box. In the unpolarized dataset, 88% of particles are consistent with the depolarized conformation, while only 34% of particles in the polarized dataset are in the depolarized conformation. The symmetry used for refinement and classification, particle counts for the selected classes and the software package used is indicated for each step.

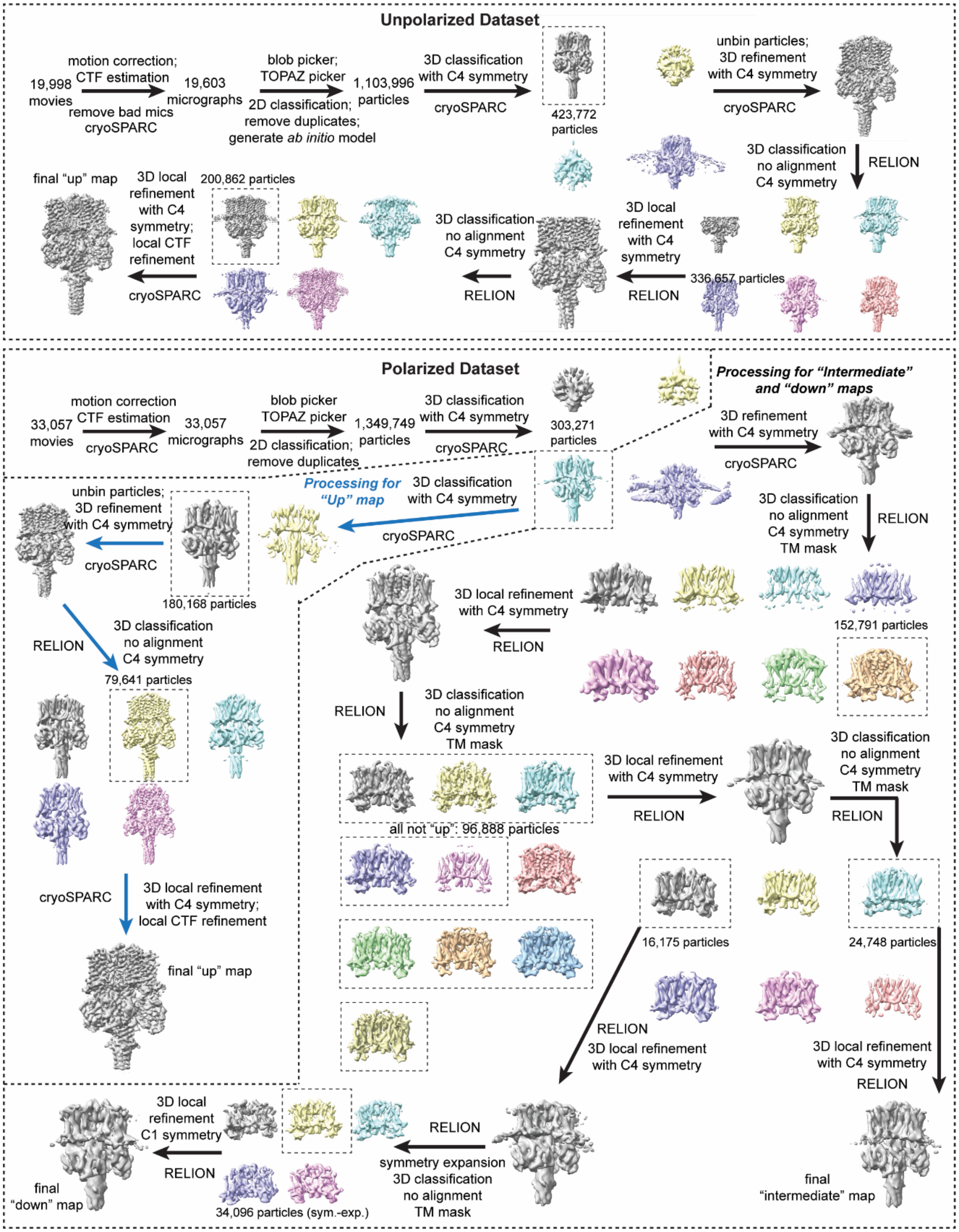

Figure S3. Workflow for final cryo-EM maps from the unpolarized and polarized datasets.

Final cryo-EM reconstructions from the unpolarized and polarized datasets. Cryo-EM maps from refinement or classification are shown in solid surface. Classes selected for further refinement are marked by a dashed grey box. The symmetry used for refinement and classification, particle counts for the selected classes and the software package used is indicated for each step.

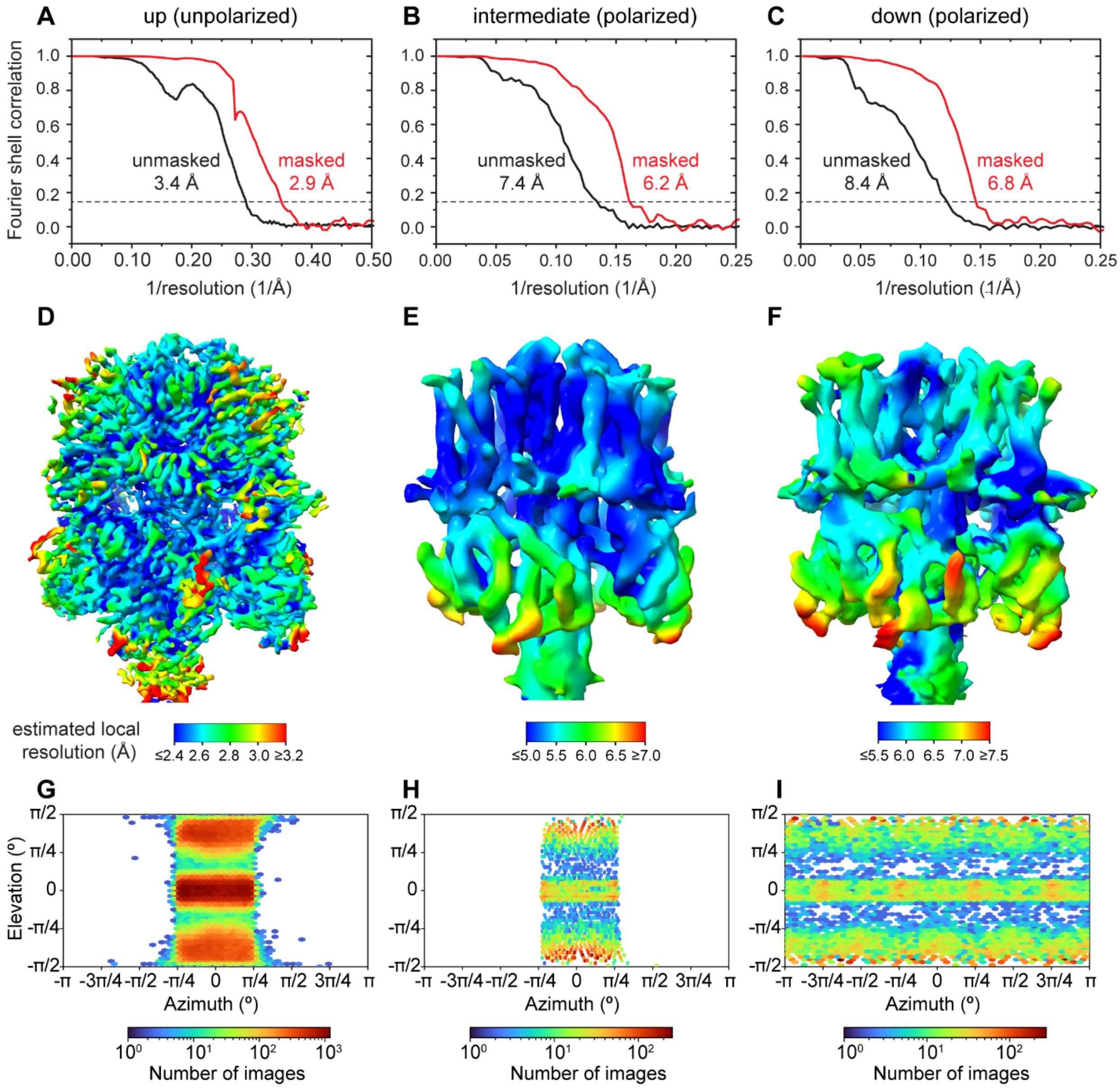

Figure S4. Fourier shell correlation curves and local resolution estimates for the cryo-EM maps.

(**A-C**) Fourier Shell Correlation (FSC) curves for the (**A**) up, (**B**) intermediate and (**C**) down maps calculated using the two independent half-maps from refinement. FSC curves for masked maps are shown in red and those for unmasked maps are in black. The nominal resolution at the gold-standard criterion (FSC=0.143, black dashed line) is given for each map. (**D-F**) CryoSPARC-derived local resolution estimates (FSC = 0.143) for the (**D**) up, (**E**) intermediate and (**F**) down maps overlaid with the corresponding maps. (**G-I)** Distribution of orientations over the elevation and azimuth angles for particles included in the calculation of the final (**G**) up, (**H**) intermediate and (**I**) down maps. The key for the heat map is included below each plot.

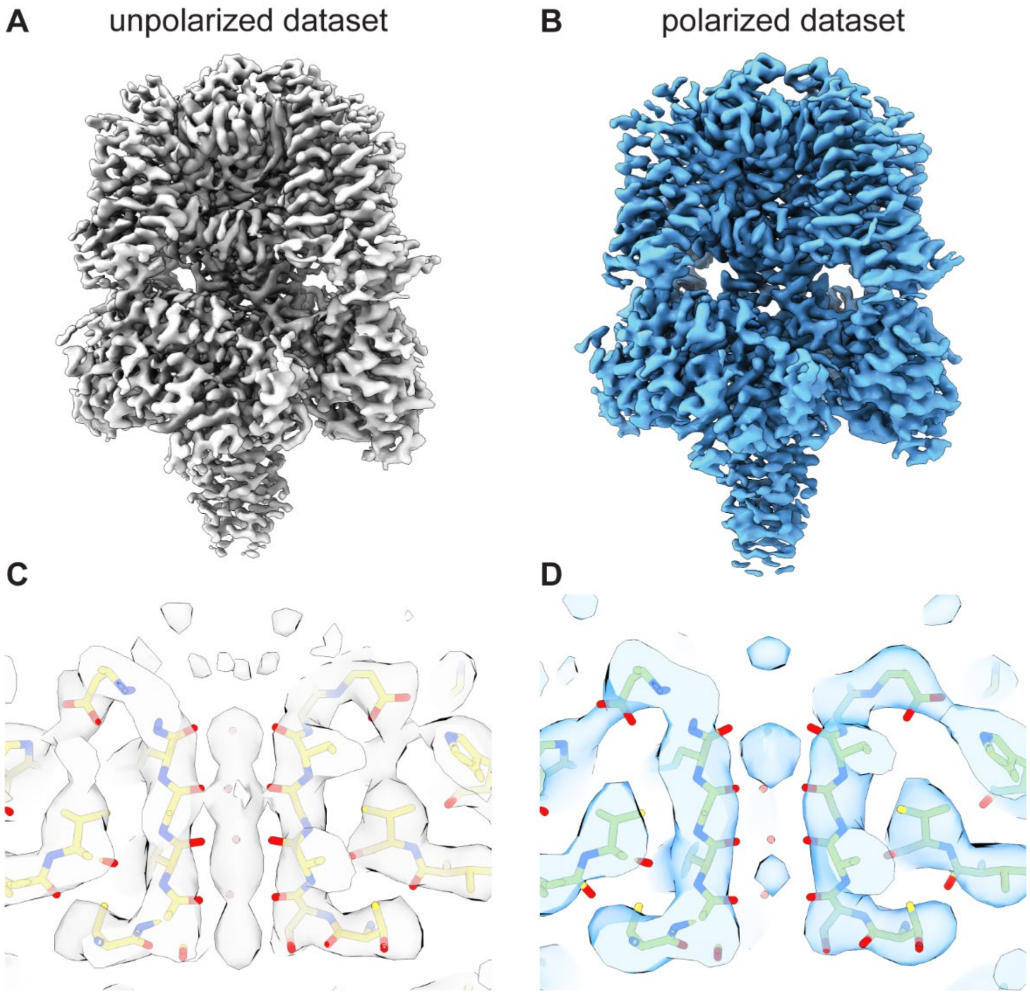

Figure S5. Comparison of up structures from the unpolarized and polarized datasets.

(**A-B**) Cryo-EM density map of the up structure of the KCNQ1 channel from the unpolarized dataset (**A**; 2.85 Å overall; 200k particles) and the polarized dataset (**B**; 3.35 Å overall; 77k particles). (**C-D**) Side views of the selectivity filter of KCNQ1 in the unpolarized dataset (**C**) and in the polarized dataset (**D**). The low external concentration of K^+ (~1 mM) in the polarized sample results in lower ion occupancy.

A

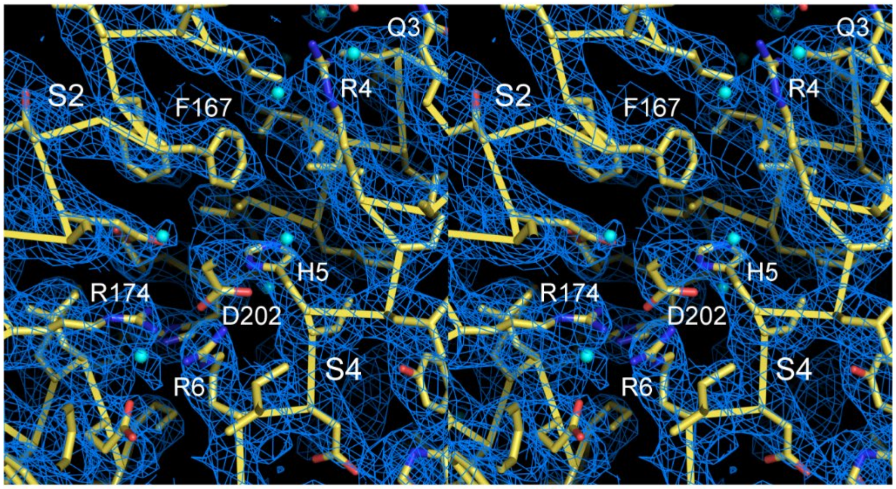

B

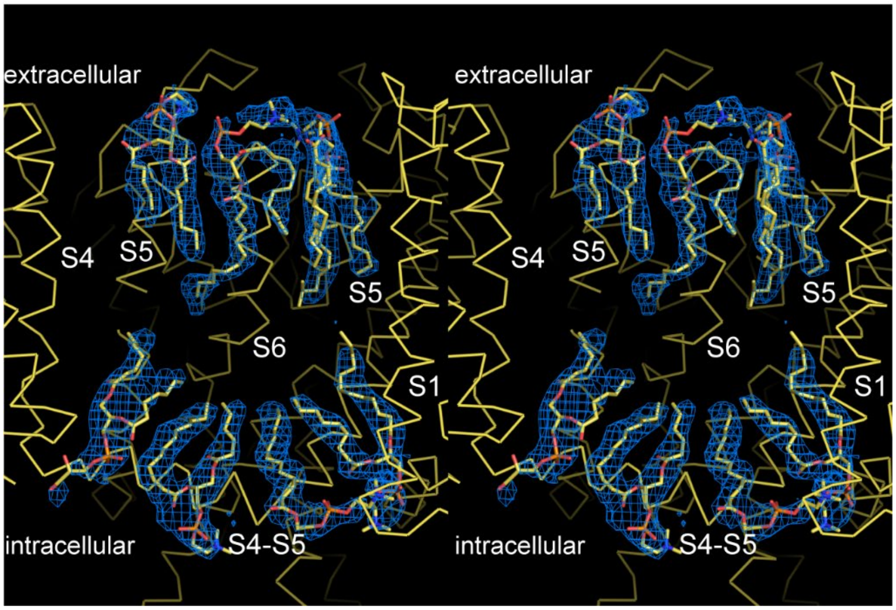

Figure S6. Water molecules in the voltage sensor and lipids in the up structure from the unpolarized dataset.

Carbon α trace (yellow) of the up model with cryo-EM density (blue) overlaid. (**A**) Stereoview showing water molecules (cyan) in the interior of the voltage sensor (shown in stick representation). The hydrophobic Phe (F167) in the gating charge transfer center, the last four positive charged residues in S4 (Q3 to R6), R174 in S2, and D202 in S3 (which is partially hidden due to the clipping mask) are labeled for reference. (**B**) Side view (stereoview) of the channel showing partial models for tightly associated phospholipid and sterol (partially obscured by a lipid) molecules (stick representation) in the outer and inner leaflets of the membrane. Helices adjacent to these molecules are labeled for reference.

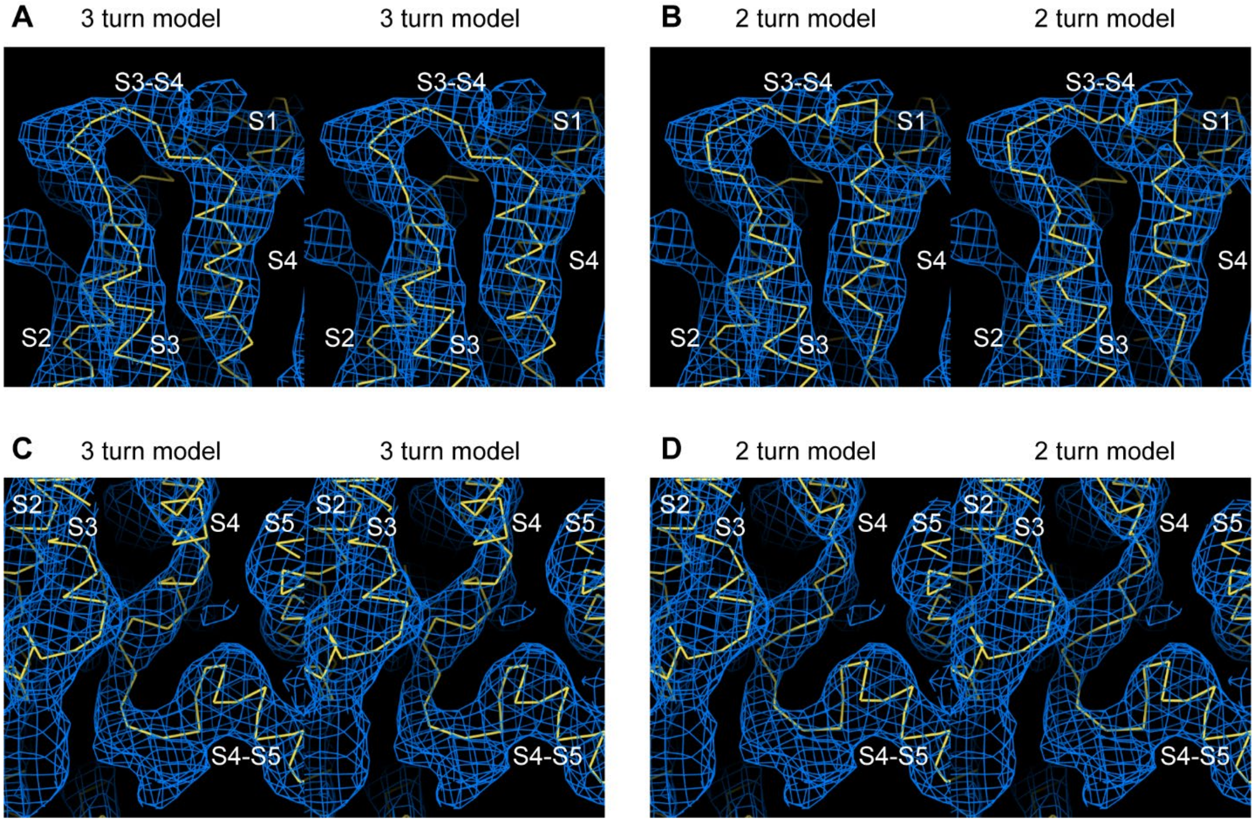

Figure S7. Comparison of 3 helical turn and 2 helical turn models for the down map.

(**A-B**) Stereoview of the top region of S4 and S3 showing cryo-EM density in the down map (blue mesh) overlaid with the 3 helical turn model (**A**) or the 2 helical turn model (**B**) shown in Cα-trace representation. (**C-D**) Stereoview of the bottom region of S4 and the S4-S5 linker showing cryo-EM density in the down map (blue mesh) overlaid with the 3 helical turn model (**C**) or the 2 helical turn model (**D**). The 3 helical turn model fits better at both the top and the bottom of S4 but the 2 helical turn model cannot be excluded.

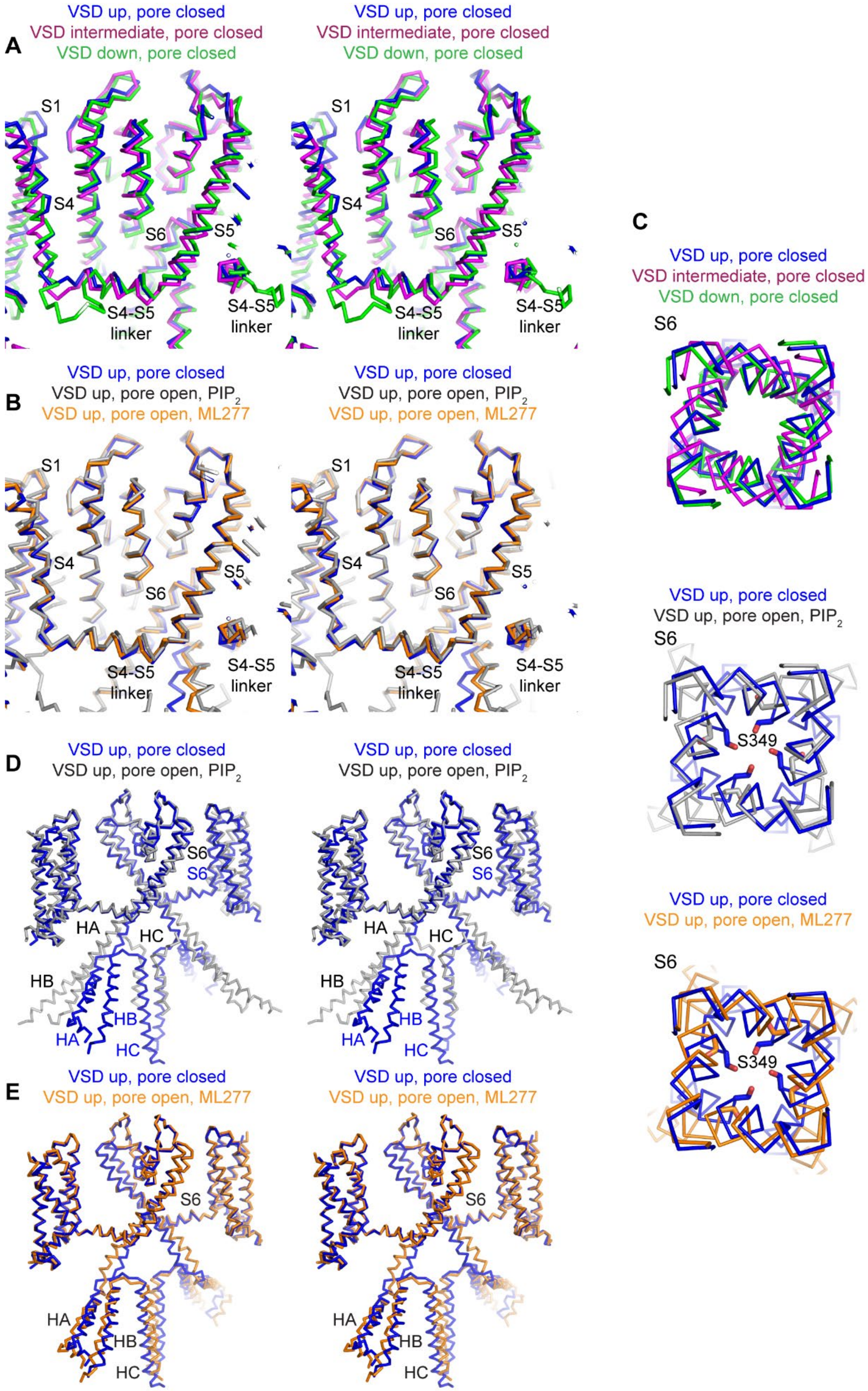
A
VSD up, pore closed
VSD intermediate, pore closed
VSD down, pore closed
S1
S4
S6
S5
S4-S5 linker
S4-S5 linker
B
VSD up, pore closed
VSD up, pore open, PIP_2
VSD up, pore open, ML277
S1
S4
S6
S5
S4-S5 linker
S4-S5 linker
C
VSD up, pore closed
VSD intermediate, pore closed
VSD down, pore closed
S6
VSD up, pore closed
VSD up, pore open, PIP_2
S6
S349
VSD up, pore closed
VSD up, pore open, ML277
S6
S349
D
VSD up, pore closed
VSD up, pore open, PIP_2
S6
S6
HA
HC
HB
HB
HA
HC
E
VSD up, pore closed
VSD up, pore open, ML277
S6
HA
HB
HC

Figure S8. Comparison of S4-S5 linker position, cytoplasmic domain (CTD) conformation and pore dilation in different structures of KCNQ1.

(**A-B**) Stereoviews showing the S4-S5 linker and the surrounding regions (Cα trace) in two adjacent subunits with secondary structure elements labeled. (**A**) The three voltage sensor conformations determined in this study (blue: up; magenta: intermediate; green: down), all with a closed pore due to the absence of PIP_2. (**B**) The up voltage sensor and pore closed conformation from this work (blue) overlaid with the up voltage sensor and PIP_2-bound pore open structure (grey; PDB ID: 6V01) and the up voltage sensor and ML277-bound pore open structure (orange; PDB ID: 7XNK). (**C**) Top-down (from the extracellular side) views of the pore (S6 helices) in the three voltage sensor conformations (top), with an up voltage sensor in the absence and presence of PIP_2 (middle), and with an up voltage sensor in the absence and presence of ML277 (bottom). S6 is shown in Cα trace and S349 is shown as stick representation. The pore remains closed in all three voltage sensor conformations, but opens when PIP_2 or ML277 (or both) are bound. All structures were aligned by their pore helices and selectivity filters. (**D-E**) Stereoviews showing two opposite KCNQ1 monomers (Cα trace) to highlight conformational changes in the CTD. (**D**) The up voltage sensor and pore closed conformation (blue) overlaid with the up voltage sensor and PIP_2-bound pore open structure (grey, PDB ID: 6V01), showing the large rearrangement in the CTD upon PIP_2-binding. (**E**) The up voltage sensor and pore closed conformation (blue) overlaid with the up voltage sensor and ML277-bound pore open structure (orange, PDB ID: 7XNK), showing the CTD conformation does not change much despite pore opening.

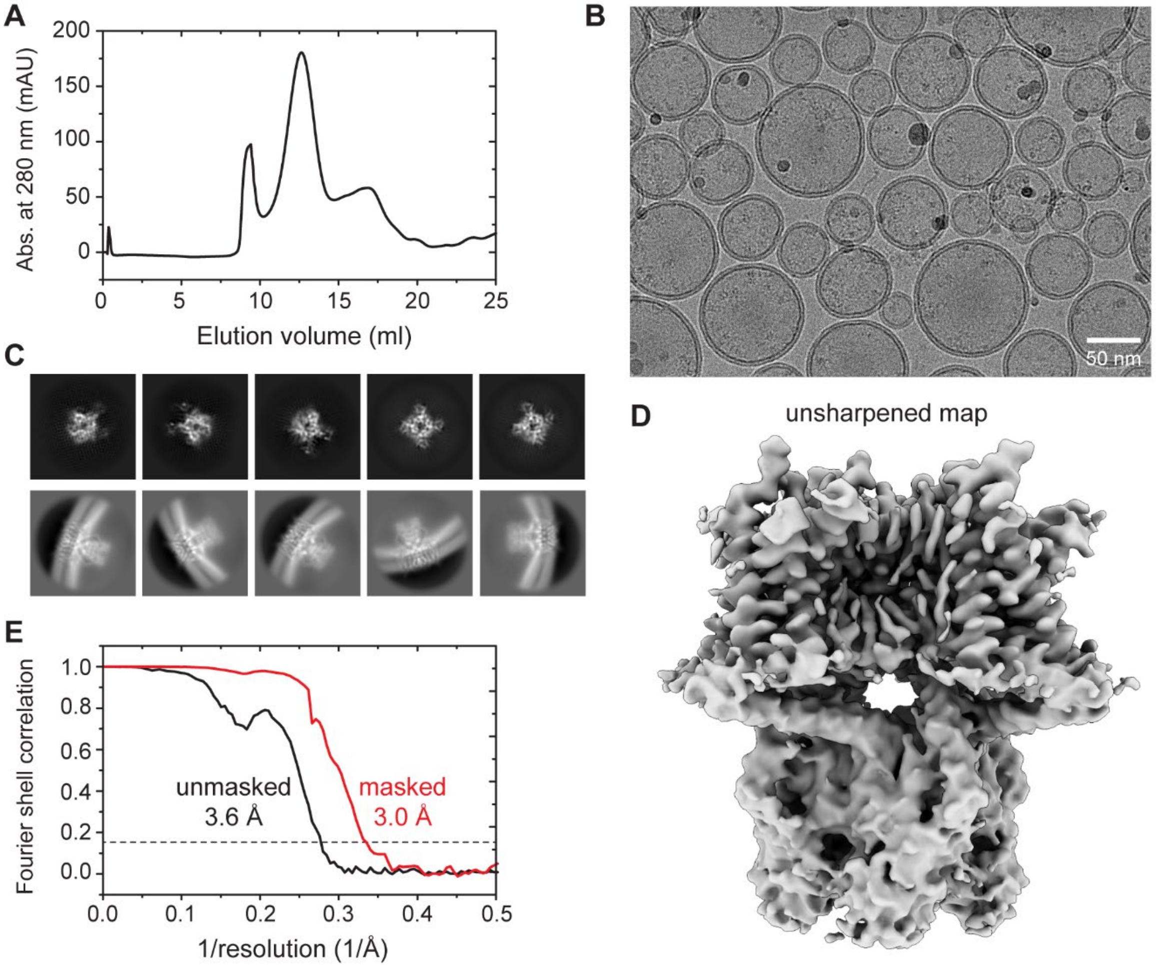

Figure S9. Purification and structure determination of hK_v2.1 in lipid vesicles.

(**A**) Gel-filtration chromatogram of hK_v2.1 on a Superose 6 Increase column. The peak at ~12.5 mL was used for reconstitution. (**B**) Representative cryo-EM micrograph showing vesicles containing K_v2.1 (scale bar: 50 nm). (**C**) Representative 2D classes showing top/bottom views (top row) and side views (bottom row) of K_v2.1 in lipid vesicles. (**D**) Final unsharpened cryo-EM reconstruction shown at a low threshold to highlight the heterogeneity of the cytoplasmic domain, while the transmembrane domain is well resolved. (**E**) FSC curves for masked (red) and unmasked (black) maps calculated using the two independent half-maps from refinement. The nominal resolution at the gold-standard criterion (FSC=0.143, black dashed line) is indicated.

Table S1. Summary of cryo-EM reconstruction and structural model statistics.

Reconstructions	Up	Intermediate	Down
Dataset	Unpolarized	Polarized	
Microscope/Camera		Titan Krios 2 300 kV / Gatan K3	
Pixel Size	0.839 Å	1.678 Å	1.678 Å
Total dose		60 $e^{-}/Å^{2}$	
Defocus range		-1.0 to -2.0 μm	
Movies collected	19,998	33,057	
Particle number	200,862	24,748	34,096
Symmetry imposed	C4	C4	C1
Overall resolution (masked)	2.9 Å	6.2 Å	6.8 Å
**Models**	**Up**	**Intermediate**	**Down**
**Ramachandran plot**			
Preferred (%)	98.36	98.31	97.57
Allowed (%)	1.64	1.69	2.43
Outliers (%)	0.00	0.00	0.00
**MolProbity**			
Clash Score	6.58	11.84	10.44
Rotamer Outliers (%)	0.90	0.00	0.00
Cβ deviations	0.00	0.00	0.00
Overall Score	1.37	1.59	1.63
**RMS deviations**			
Bond lengths (Å)	0.003	0.002	0.002
Bond angles (°)	0.473	0.593	0.519

Movie S1 (separate file). Comparison of cryo-EM density in the up and down structures.

This movie shows side-by-side views of the lowpass filtered up structure (left) and the down structure (right). The protein is shown as a blue Cα trace with S4 highlighted in red. First the up structure is rotated and then the down structure to show the difference in density between the two maps.

Movie S2 (separate file). Sequence of conformational changes occurring during channel gating.

The movie shows a side view of the morph between three structures of KCNQ1: with the pore closed and voltage sensor down (this work), with the pore closed and voltage sensor up (this work), and with the pore open and voltage sensor up (ML277 bound, PDB ID: 7XNK). The protein is shown in Cα trace representation and PIP_2 is shown in yellow stick representation. The S4 and S6 of one subunit are colored blue and green, respectively. At hyperpolarized membrane voltages (i.e. the resting potential of a cell), the voltage sensor is in the down conformation, which prevents PIP_2 binding. Depolarization drives the voltage sensor up, which then allows PIP_2 to bind and promote opening of the pore. The sequence then runs in reverse. Note that the pore open and voltage sensor up structure with PIP_2 bound (PDB ID: 6V01) was not used for the morph because it contains KCNE3, which causes a rotation of the voltage sensor relative to the pore and distracts from the pore opening we wish to show.
